# Lectures on the Physical Phenomena of Life, Delivered at the College of France

**Published:** 1839-10

**Authors:** 


### BRITISH AND FOREIGN MEDICAL REVIEW,

FOR OCTOBER, 1839.

### PART FIRST.

### Analytical and Critical Reviews.

### Art. I.

*Leçons sur les Phénomènes Physiques de la Vie, professées au Collége de France.* Par M. Magendie. Recueillies par C. James et G. Funel. —*Paris,* 1836-8. 4 Tomes, 8vo, pp. 310, 376, 471, 400.

*Lectures on the Physical Phenomena of Life, delivered at the College of France.* By M. Magendie, and collected by C. James and G. Funel.—*Paris,* 1836-8. Four Vols. 8vo.

In the writings of most physiologists, from the earliest times to the present, one of two very opposite but equally erroneous tendencies may be observed. While some have regarded the phenomena of life as resulting, in all instances, from the operation of laws entirely different from those which influence inert matter, others have as exclusively resorted to the various branches of physical science for the solution of phenomena which these are utterly inadequate to explain. Now it requires but a moderate degree of observation to become convinced that, although there are powers at work in the living organism, which produce results quite different from any presented in the inorganic kingdom, and which we may therefore reasonably presume to be distinct and peculiar in their nature—many processes are nevertheless carried on, which, though conducive to vital ends, and under the control of the hidden powers of life, are, in their immediate nature, purely physical. However obvious this truth may appear when clearly stated, it has in general been absent from the speculations of physiologists; so that although a host of ingenious minds have been occupied in bringing physical laws to bear on vital phenomena, which such laws cannot explain, few have cared to apply them to a large range of phenomena in the living body, which they can explain, and which in truth are explicable in no other way.

To distinguish the *physical* phenomena of living bodies from the *vital*, and to illustrate the former, and refer them to the laws from which they result, are the professed objects of M. Magendie in the course of lectures before us; and these objects are stated, at the commencement, with sufficient precision. Judging from the author's reputation as a physiologist, the knowledge of chemistry evinced in some of his writings, and his experience in the practice of medicine, it might be thought that the subject could not have fallen into better hands. In the progress of the present work, however, we meet with too many proofs that a man may possess great ingenuity and considerable acquirements, with very small capacity —we had almost said, a total incapacity—of sustained reasoning. Led astray by a predominating idea, M. Magendie continually loses sight of the distinction which he admits as the basis of the enquiry, and deviates from the path he has himself prescribed. Instead of carefully separating the physical phenomena of life from the vital ones with which they are so intimately associated, his chief anxiety seems to be to make out as many physical and as few vital as possible.

The mode of investigation he adopts is much better calculated to strike an audience with momentary conviction than to enlarge the real limits of science. It consists chiefly in experiments, by which it is made evident to the senses that certain phenomena take place under certain circumstances; but little pains are bestowed to ascertain what would have taken place under other circumstances. The fact auspicious to the preconceived view is seized upon, and a hasty generalization set on foot. Indeed, we find ourselves obliged to declare, that we know not, in the whole range of modern scientific literature, a work which contains so frequent violations of the inductive principle, or such rash generalizations from isolated facts, as that before us.

Independently, also, of the unphilosophical manner in which the inferences are drawn, we must express our distrust of the source from which a great many of the data are derived; namely, destructive experiments on living animals. We feel convinced that an animal mortally injured, in which every action of life is fast ebbing and the organ immediately experimented upon placed *suddenly* in circumstances incompatible with the continuance of its functions, can, generally speaking, afford no just solution of the phenomena of life, whether vital or physical. If this objection be deemed too vague and general, as we are aware it may, at first sight, we appeal at once to experience, as to the results of such methods of enquiry; and we ask how it is, if they be worthy of the confidence which some have reposed in them, that the ablest physiologists are continually at variance as to the *facts* elicited from them, one thing being observed by one experimenter, and something quite different, or diametrically opposite, by another? We do not at all deny that there are instances in which such experiments are of the utmost utility, and afford the only means of determining a question; but, as a general *basis* of physiological research, we believe them to be entirely delusive. Again, no respect is paid by M. Magendie to the physiological differences of animals; and an inference from the changes which ensue in the body of one animal is made applicable to the whole class, as well as to man.

The perusal of these volumes, indeed, confirms an opinion which we had formed from a knowledge of the previous labours of this physiologist; that, whatever tact he may possess in performing experiments, he is not well qualified to draw conclusions from the results. Thus we find him repeatedly deducing an inference from certain appearances in one lecture, expressing a dogmatical opinion off-hand, and coolly retracting that opinion in a subsequent lecture. There may be candour, in thus publicly acknowledging an error, but we must contend that such hasty conclusions betray a great want of judgment, and serve to create distrust in the opinions of the experimentalist. Many years ago, M. Magendie startled all sober physiologists by the assertion that the grand sympathetic was not to be considered as a part of the nervous system of animals; and that he had removed the cervical and superior thoracic ganglia, without causing the least disturbance in the animal functions. (*Précis Elément. de Phys.* tom. i. p. 172, 1825.) In the work before us we see the same spirit displayed in numerous instances. The same want of caution, the same hasty generalization, the same indifference to strictly inductive reasoning, exist—the subject only is changed, not the mode of investigation. The result is what might be anticipated.

We have premised these general remarks, in order to avoid needless repetition, and now proceed to consider the contents of these lectures in detail. We shall, as much as possible, take up the different subjects in the succession in which we find them; but as there is throughout a total want of logical arrangement, with its necessary consequence of frequent iteration, we shall not hesitate to make such transpositions as perspicuity or condensation may demand.

*Porosity and Imbibition.* Every substance in nature is more or less porous, and the organic textures are endowed with porosity in various degrees. All porous bodies present the phenomenon of capillary attraction, and to this, as manifested in the organic textures, M. Magendie gives the name of *imbibition*. Our author makes imbibition the key to the whole doctrine of absorption. The following is a summary of the conclusions, with reference to this function, which he assumes as established by his former researches, or deduces from experiments presently to be detailed.

In every instance of absorption there are two distinct phenomena, which we should be careful not to confound. 1st. The introduction of a liquid by imbibition. 2d. Its conveyance into the current of the circulation. The lymphatic vessels have nothing to do with absorption, which is exercised solely by the veins, except in the instance of the absorption of chyle, which is effected by the lacteals. M. Magendie contends against the opinion that the absorbing surfaces of living bodies select the substances fit to be absorbed, and reject the rest: he maintains that they take up substances on the simple principle of capillary attraction, and hence that the facility with which substances are imbibed, depends entirely on their physical properties—soluble substances being more readily taken up than insoluble ones, and volatile substances more readily than those which are fixed: from all which it would appear, that the vital function of absorption, as hitherto regarded by physiologists, has no existence; the phenomena referred to it being purely physical. Imbibition exists in various degrees, in the different organic textures. It is greatest in the serous and cellular membranes, less in the blood-vessels, less still in the mucous membranes, and least in the dermoid textures. The epidermis, under ordinary circumstances, may be said not to imbibe at all; nevertheless, if a substance apt for imbibition be applied to it for a length of time, it is absorbed. Though the imbibing power of any two surfaces be equal, the transit of substances through them into the system will be more or less speedy, according to their degree of vascularity; thus the pleura and peritoneum are both serous membranes, and as such may be supposed to imbibe in an equal degree; but the pleura is the more vascular, and hence a poison injected into the cavity of the thorax will act on the system more rapidly than if it were injected into the cavity of the abdomen. Active substances do not affect the system through the medium of the nerves, but by entering the circulation; the rapidity, therefore, with which such substances produce their effects, is exactly proportioned to the facility with which they are imbibed, and the vascularity of the imbibing organ. The following are apt illustrations of imbibition.

If we put a drop of an aqueous solution of iodine on a sheet of paper, the colouring matter remains in the centre, and the more liquid part is diffused towards the circumference. Suppose, now, that a man has received a severe blow on his arm; blood is effused, and an ecchymosis formed, occupying a limited space. What do we observe the day after the accident? The contused point is black, while the neighbouring integuments are of a yellow colour, which often extends as far as the shoulder and fore-arm. In fact, the black part of the blood remains where it was first effused, and the serum, charged with yellow colouring matter, is infiltrated into the textures. What is there of vital in all this? We cannot suppose that the absorbents have conveyed the colouring matter; for, since the imbibition takes place below as well as above the injured part, their action would then be retrograde. (Vol. i. p. 20.)

Again, an aqueous solution of the ioduretted iodide of potassium is injected into the abdominal cavity of a rabbit. On laying open the abdomen, the serous coat of the intestine is found to be penetrated and coloured by the injection, the liquid having been imbibed through the parietes of the vessels. We must not wait too long before opening the abdomen, or the whole of the liquid will have disappeared, and been taken into the circulation. If a few drops of the liquid be placed upon the stomach, a similar phenomenon is observed. (Vol. i. p. 21.)

Before going further, we must enter our protest against the arbitrary manner in which M. Magendie assumes it as proved that the lymphatics have no part in the function of absorption. This is one of many instances in which, having convinced himself, he thinks that every one else ought to be convinced also. It would be out of place here to enter into a discussion of so disputed a point, but we may be allowed to ask, if M. Magendie's experiments be so decisive, how is it that his conclusions are not generally received? He must be aware that the greater number of contemporary physiologists, while they fully admit that he has established the fact of absorption by the veins, still believe the lymphatics to bear a part, and a principal part, in the function in question. While it is the attribute of genius to conceive new ideas, and to devise new modes of research, the validity of the deductions derived must ever be determined by the impartial judgment of others.

We do not positively assert that M. Magendie's doctrine is erroneous, though we believe it to be so; but we affirm that, in the present state of our knowledge, M. Magendie has no right to assume, as a basis of physiological reasoning, that the veins are the sole absorbents. Neither can we admit that the function of absorption is, in all instances, dependent on the common laws of physics, although M. Magendie has shown that it is so in some cases, where it might formerly have been regarded as a vital process.

We may adduce one or two examples of the inapplicability of M.

Magendie's hypothesis. It is well known that the cavity of a circumscribed acute abscess is lined with a dense membranous substance, much resembling a layer of organized lymph; and it is further well known that abscesses have a remarkable and salutary tendency to open on the surface of the body, without which they would always endanger life, when situated near any of the great cavities: now, can M. Magendie tell us what change is effected in the physical properties of the more superficial portion of the sac that should render it more easy of absorption than any other portion? In the course of these lectures, M. Magendie remarks on the bursting of an abscess, that it is partly by the successive imbibition of the pus into the cellular tissue, that the skin is penetrated throughout its layers, grows thin, and finally gives way, (vol. i. p. 106;) but he does not inform us why the pus at first made its way through the firm parietes of the abscess at one point rather than another. We suspect this looks very like an increased action in the absorbent vessels themselves, set up by the instinctive power of the living organism. Again, when sphacelus has taken place in a part, and a line of separation is drawn between it and the living parts, and the intervening substance is absorbed so as completely to detach the slough, can M. Magendie show us any physical change in the particles taken up which renders them more apt for imbibition than they were before? or can he tell us why this separation of the mortified part happens in one case and not in another, the physical condition of the dead and living parts being, in the two cases, to all appearance exactly analogous? While we are unsatisfied on these heads we must remain under the conviction that the absorbents do actually exercise a vital selection, and that the reason why a mortified part is separated in one case and not in another is that the vital powers are sometimes equal to the effort, and sometimes not.

A third point on which we must differ entirely from M. Magendie is that of cutaneous absorption. He denies that any absorption takes place through the cuticle under ordinary circumstances. Now, considering the direct evidence in favour of cutaneous absorption to be found in the experiments of various physiologists, and especially in those of Dr. Milne Edwards, which are generally thought to be conclusive,* we expected some new and very striking facts from M. Magendie on the opposite side of the question. Nothing, however, is adduced, but that he has often received on his own skin, the saliva of persons labouring under hydrophobia, without any bad result! We can attach no importance whatever to this fact; for although, according to M. Magendie's experiments, the disease may be communicated to the dog by inoculation with the saliva of a hydrophobic person,† it is entirely unknown whether it be communicable from one human being to another, under any circumstances, the susceptibility of the human subject being comparatively very small. It is equally unknown whether the virus acts through the medium of absorption, or by direct application to the extremities of the nerves; and even adopting the former supposition, a viscid liquid, like the saliva

* We do not mention the experiments of M. Collard de Martigny, because they are of a date posterior to that of the lectures under review. For an account of these and others, see the review of Dr. Madden, Br. and For. Med. Rev. vol. VI. p. 332.

† It were to be wished that these experiments had been repeated by some other observers.

of a hydrophobic patient, applied in small quantity and drying immediately on the surface, is not likely to be readily absorbed through the cuticle, the imbibing power of which is acknowledged to be only of moderate intensity.

We may now consider some of the principal applications which M. Magendie makes of his peculiar doctrine of absorption.

*The Mode of Operation of Poisons.* Some drops of an alcoholic solution of nux vomica are injected into the pleural cavity of a rabbit; the animal is instantly seized with tetanus, and dies. In another experiment, a small arrow, envenomed with the alcoholic extract of nux vomica, is driven into the thigh of a rabbit: five minutes elapse before any sensible effect is produced. The diversity of result in these two experiments arises, according to M. Magendie, from two causes, viz., the poison being solid in the one instance and dissolved in the other, and the greater vascularity of the pleura than of the muscular substance of the thigh. If, at the moment when the rabbit into whose thigh the poison has been inserted begins to experience its effects, the member be strongly compressed with the hand, the influence of the poison is suspended; but the moment the compression is removed, the animal falls dead. From these and similar experiments, M. Magendie deduces his opinion that the rapidity with which poisons produce their effects is in the exact ratio of the facility with which they are absorbed, and that the circulation is the only medium through which they operate. We cannot help thinking that if he had made a careful comparison of the physical properties of the different active poisons, he would have hesitated to adopt such a belief.

Hydrocyanic acid, the most rapidly fatal of all poisons, doubtless affords an instance favorable to M. Magendie's hypothesis, for it is a highly attenuated and volatile substance. But let us try strychnia. In an experiment of Orfila's, half a grain of this substance introduced into a small incision made in the back of a rabbit caused convulsions in one minute and death in three and a half.* It is to be observed that the question before us is not as to the energy of the poison in destroying life, but simply as to the celerity of its effect. Now strychnia is so insoluble in water, that it requires about 6667 parts of cold and 2500 of boiling water to dissolve it; and it is volatile only at temperatures so high as to decompose it: its properties are therefore entirely unfavorable to speedy imbibition; and we doubt much whether it be possible that, in the experiment alluded to, a quantity of the poison sufficient to produce its specific effects should have been conveyed into the circulation in a minute. According to the experiments of Müller, an appreciable quantity of a substance *in solution* brought into contact with a membrane not covered by epidermis, may be distributed through the system by means of the blood-vessels in from half a minute to two minutes;† but in this experiment with strychnia, the substance is *solid* and eminently insoluble.

Again, take the poison of the rattle-snake. Captain Hall saw a dog killed by the bite of this animal in fifteen seconds, and another in thirty seconds.‡ The effects of the bite of this serpent are not generally so

* Traité des Poisons, tom. ii. p. 372. Paris, 1826.

† Elements of Physiology (Baly's translation), pp. 245-6.

‡ An account of some experiments on the effects of the poison of the rattle-snake. By Captain Hall. Communicated by Sir Hans Sloane. Philosophical Trans. for 1727.

rapid, but the observations seem to have been very carefully made, and the more than usual energy of the poison does not render the instance less available for our purpose, which is to show how quickly a poison may act on the system. Now, if M. Magendie's opinion were correct, this poison should be a very penetrating and volatile substance. But it appears from the observations of Dr. Russell that the poisons of venomous serpents, which are all remarkably similar in their physical properties, consist of a mucilaginous or glutinous liquid, of a yellowish colour, devoid of pungency, and insipid, readily soluble in water and alcohol, when fresh, but soon drying into a yellow, flaky, resinous-looking substance, which grows darker coloured when long kept, and is then not easily soluble.* An exact analysis of these poisons is still a desideratum, and, for anything we know, they may contain some highly subtile and volatile principle, but from all that is ascertained—which is all that anybody has a right to reason from—there is nothing in the physical properties of such poisons to account for any remarkable rapidity of effect on the score of ready imbibition. These few examples may suffice to show that M. Magendie's conclusion is at least premature, and that no attempt should be made to base any theory of the operations of poisons on their physical properties till these properties have been subjected to a careful and extensive examination. The confidence with which M. Magendie asserts the operation of poisons through the exclusive medium of the circulation perfectly astounds us, when we consider the extreme difficulty and complexity of the investigation, the great discrepancy of opinion among the best physiologists of the day, and the countenance afforded to an opinion very different to M. Magendie's by the careful and well-devised experiments of Morgan and Addison. The inference from the effects of compression on a limb is of little weight, because supposing the poison to operate on the nerves, these, like other organs, require a due supply of blood for the maintenance of their functions; and hence it is not surprising that no impression should be made on them when they are not in a condition to be impressed. Moreover, a similar arrest of action may be caused by compression where there is no poison in question: has not an *aura epileptica* often been arrested, and a fit averted, by a tight ligature round a limb? Yet no one supposes that in this case any poison enters the veins: the presumption is that a morbid action which has commenced in the extremity of a nerve, and would have been propagated along its course, is stopped by cutting off the supply of blood.

On the whole, M. Magendie's experiments on the absorption of poisons do not seem at all to warrant the conclusions he derives from them. They merely tend to prove that, *cæteris paribus*, the more easily poisons are imbibed, the more rapid will be their effect.

*Contagious Diseases.* The real number of these M. Magendie regards as very small; and he flings equal contempt on governments and doctors who would extend the list. When speaking of the discrimination of truly contagious diseases from those which are only reputed to be so, he asks, " Is it not an afflicting thing to see that these questions, essentially belonging to the province of medicine, have been resolved by men

* Experiments on the Poisons of several Indian Serpents, &c., p. 86.

strangers to this science; and that our medical legislation still rests on the most erroneous assertions? Thus the law recognizes five contagious diseases, and punishes with death every individual who infringes the rules laid down to prevent their introduction. Well; of these five diseases, four at least ought to be erased from the list." (Vol. i. p. 64.) The four diseases alluded to are typhus, cholera, yellow fever, and leprosy, "the contagion of which," says M. Magendie, "is positively denied by every enlightened physician." Now, we would ask, in our turn, is it not afflicting to see such a man as M. Magendie, who is no stranger to medicine, and who takes his ground as a philosopher, hazarding an assertion at once so rash and so illiberal, as that no enlightened physician believes typhus or cholera to be contagious? We humbly hope that the profession is not altogether benighted in this country, yet nineteen English physicians out of twenty, and probably a much larger proportion, firmly believe typhus to be contagious; and a considerable number are of the same opinion with respect to cholera. As to typhus, we must confess that we are ourselves shrouded in thick darkness; for we feel just as certain that typhus is contagious as that the gout is not. On the contagious nature of cholera we are in a state of twilight: being averse to hasty conclusions, we have not yet made up our mind; but it suffices for our argument that the disease is considered contagious by many men eminent both as physicians and philosophers.

There is in general little to interest in our author's remarks on the diseases reputed to be contagious. The following fact, however, is worthy of notice: M. Magendie has found, by experiment, that if a few drops of water, impregnated with putrid meat or fish, be introduced into the sanguiferous system of a dog, the animal first presents an unusual degree of activity, then is affected with fever, lies down, refuses food, and vomits an immense quantity of the black matter characteristic of yellow fever. (*Ib.* p. 67.) In another place he states that in similar experiments petechiæ on the skin were also produced. (*Ib.* p. 117.) This observation, interesting in itself, is followed by one of those sweeping affirmations too common with our author, "We know also," says he, "that in all circumstances under which yellow fever is developed, the air has been vitiated and corrupted by the disengagement of putrid animal products. Thus it is not rare to see it break out when a vessel laden with cod runs aground in the neighbourhood of a town, and the merchandize (fish) being heaped up, exhales into the atmosphere infectious miasmata. Let these corrupted matters be thrown into the sea, and the disease will soon disappear. Thus the cause which produces and keeps up yellow fever being perfectly known, we have only to guard against it in order to have nothing to fear from this terrible scourge!" (*Ib.* pp. 67-8.) This certainty in the etiology of yellow fever is quite new to us. Since writers of the highest authority, and who have had the most ample opportunities of observation, have differed entirely on the subject, some referring the cause of this disease to imported contagion, some to marsh miasm, some to animal putrefaction, while others, who have had the advantage of preceding opinions in addition to their own experience, declare frankly that they do not know what the cause is, we had surmised that there must be great difficulty in settling the question; but M. Magendie soon gets rid of a difficulty!

Our author speaks at some length on the subject of plague. He doubts whether it be contagious, or communicable through the medium of fomites; but supposing it to be so, he is convinced that the notion of its being communicated by contact with the diseased body must be erroneous, and that the real mode of communication is by pulmonary absorption, which, he contends, is the medium through which all contagious diseases are propagated. He says that the itch forms no exception to this rule, since it is not propagated by a virus but by an insect. We will not stop to contest this point, though there is good ground; but may suggest, in passing, the instance of *porrigo scutulata.* Is this not contagious? There are few diseases more so. Is it also propagated by an insect? The assertion, we believe, has never been made. With respect to plague, M. Magendie asks, "by what singular exception to all the known laws of imbibition is it that the skin, protected by the epidermis, possesses such strong absorbent powers, while the pulmonary surface is completely destitute of them? This is opposed to every precept of experience and reason." (Vol. i. p. 72.) But he seems here to be contending against a doctrine which no one maintains. As far as we know, it is generally admitted by those pathologists in the present day who regard the plague as contagious, that it is very readily communicated by inhalation of the breath of the sick; and although it is maintained by many authors that contact, or a degree of proximity approaching to it is necessary to infection, this opinion, we apprehend, has reference not so much to the particular surface by which the poison is admitted into the system as to its limited diffusion, which renders a near approach to the body of the patient essential to its activity. Nor do we see any reason to deny that the poison may be admitted through the skin; for, notwithstanding M. Magendie's assertion to the contrary, there seems little reason to doubt that various attenuated substances are very easily absorbed through the skin; and if so, why may not the miasm of the plague? Lastly, we would suggest that (admitting the contagion), since the agent by which it is communicated entirely eludes our senses—since we know nothing whatever of its nature, and can only conjecture even that it is some form of ponderable matter—we have no ground of certainty that it acts by absorption at all, however probable the supposition may be that it does so. M. Magendie attacks the present system of quarantine with great vehemence, and surely not without reason; but this subject being foreign to the one immediately before us, we shall not enter into it.

*Exhalation.* We have hitherto been treating of imbibition only as it takes place from the exterior to the interior of vessels; but the same phenomenon occurs in an inverted manner, the liquids passing from the inner to the outer surface of the vessels. It is then termed *exhalation,* of which we have a remarkable and continual example in the transpiration from the lungs. There are some substances which, when introduced into the blood, cannot remain there long, and are speedily expelled, as ether, camphor, and phosphorus, which have scarcely passed into the circulation, ere their presence is detected in the vapour exhaled from the lungs.

*Endosmosis and Exosmosis.* Imbibition may take place simultaneously from within outwards, and from without inwards, thus causing a double current. M. Dutrochet, having placed a bladder filled with a liquid in another liquid of a different nature, found that, according to their different composition, the liquid contained in the bladder passed out through its parietes, or the external liquid passed in. These phenomena he named *exosmosis* and *endosmosis.* According to the physical properties of the liquids, a greater quantity of the one or the other will be transposed; generally speaking, the more viscid liquid will attract the other, but in every case there is in reality both endosmosis and exosmosis, or, in other words, a certain quantity of one fluid passes in, and a certain quantity of the other passes out. M. Magendie, therefore, proposes to call this process *imbibition by a double current* (imbibition à double courant).

The phenomena of endosmosis and exosmosis must be influenced in a variety of ways by the physical and chemical constitution of the fluids, as well as of the solids which they permeate. M. Magendie does not enter into the consideration of all the causes which determine the current in one direction rather than the other, and concludes, judiciously, we think, that in the present stage of our knowledge, they cannot be accurately explained—an opinion which coincides with that of M. Dutrochet himself, as most recently expressed.* The following is a neat experiment of M. Magendie illustrating imbibition by a double current. Take an egg, and remove a portion of the shell carefully, so as to denude the outer membrane; then place the egg in a vessel containing a little alcohol, having previously pierced a hole in the end which is not immersed. Here the results of double imbibition present themselves: the alcohol passes in by *endosmosis,* through the membrane, and coagulates the albumen, which passes out at the hole at the end of the egg, while by *exosmosis* the liquid albumen transudes through the membrane, and mixes with the alcohol in the vessel, rendering it turbid, and forming albuminous flakes. (Vol. i. p. 99.)

M. Magendie now proceeds to apply the doctrine of imbibition to the elucidation of disease. Having recapitulated the instance of a bruise, formerly cited, and instanced a leech-bite, the phenomena consequent on which admit of a similar explanation, he alludes to the subject of *encysted dropsy.* Tumours of this kind consist in a collection of liquid enveloped in a membranous bladder. It is important to attend to the nature of the contained liquid, for it is often so viscid that it will not flow through the canula of a trocar. The spontaneous cure of encysted dropsies is extremely rare; they slowly increase in size, and finally prove fatal through the impediment which they offer to the principal functions of life. We find here united the principal conditions of endosmosis: we have a bladder filled with a liquid immersed in other liquids of a different nature. It is to be observed, also, that these tumours are more difficult of cure in proportion as the liquid they contain is more viscid. Going on this principle, M. Magendie has attempted the radical cure of such diseases by modifying the nature of the fluid secreted by their internal surface. Several years ago he had under his care at the Hôtel Dieu a woman with a tumour of this kind in the ovary. By its bulk it impeded respiration and digestion. The patient was daily wasting away, and submitted

* Cyclopædia of Anatomy and Physiology, Art. *Endosmosis.*

to an operation as affording the only chance of recovery. M. Magendie made a puncture, with a view of exploration, and a viscid liquor escaped in a thread-like stream through the canula of the trocar. The tumour being emptied, some warm wine, diluted with half its quantity of water, was injected into the cavity, and, having been allowed to remain a few seconds, the greater part was drawn off. In consequence, no doubt, of the excitement produced by the injection, the tumour filled again with extreme rapidity, so that on the following day it had resumed its former volume. Another puncture was made, and the liquid, being much less viscid than formerly, flowed much more freely. The tumour again filled with serum, but by degrees diminished, and finally disappeared. M. Magendie has since repeated the same practice in two similar cases: in one he obtained the like success; in the other the tumour reappeared in spite of numerous punctures, and he was obliged to leave it to itself. In these cases M. Magendie's object was so to modify the action of the exhaling surface, as to render the liquid less viscid, and therefore more easy of imbibition through the parietes of the sac. He does not affirm positively that this was the means through which the cures were effected: his view, however, is ingenious, and accords well with his doctrine of imbibition. (Vol. i. pp. 103-5.)

A good instance of imbibition through the walls of blood-vessels is presented by an aneurismal sac, in which the concentric layers of coagulated blood are softer near the centre, and of firmer consistence towards the circumference, because the aqueous portion of the blood has been imbibed through the parietes of the vessel. (*Ib.* p. 107.)

We do not quite agree with our author when he declares that the treatment of dropsy is altogether empirical, and that hence each physician has his formula; one bleeds, another purges, and a third gives diuretics; while those who wish to conciliate different opinions, employ these several means in conjunction. (*Ib.* p. 107.) We freely admit that the treatment of dropsy is for the most part very ineffectual, but this, we think, is not so much for want of rational indications, or the means of fulfilling them, as from the too frequent presence of a perpetual cause of the disease in some organic affection of the viscera. Let us see what light M. Magendie throws on the subject.

"Dropsies (says he) are in a great measure dependent on physical laws, and may be produced at will in a living animal by determining the conditions which give a preponderance to exhalation over absorption. Every obstacle to the venous circulation is attended with serous infiltration of the parts whose veins, destined to carry the blood towards the heart, are obliterated. But there are dropsies which cannot be attributed to these mechanical causes; thus ascites sometimes exists without any appreciable alteration in the system of the vena portæ. A careful enquiry into the composition of the blood in these cases of serous infiltration, independent of any mechanical obstacle to the circulation, would afford a study at once attractive and new. It is already known that the urine is charged with albumen: this observation is interesting, but we ought to go further. Do you think that it is indifferent, with reference to the equilibrium between absorption and exhalation, whether the blood which runs through our vessels be more or less viscid, or contain more or less of the watery element?" (Vol i. p. 108.)

All this is very true; but we can see nothing peculiar in M. Magendie's pathology of dropsy, except the omission of some causes of it which are generally recognized. Pathologists, in this country at least, fully admit the mechanical obstruction of the venous circulation as a cause of diminished absorption, and a watery crasis of the blood, accompanying a leucophlegmatic habit, as a cause of increased exhalation, and both these, consequently, as causes of dropsy; but they add inflammation of serous membranes as a certain and well-known cause, while some consider as probable, though not ascertained causes, a simple increased action of the exhalents, or a diminished action of the absorbents.

M. Magendie remarks that imbibition has much to do in the formation of the various morbid products which come under the cognizance of the pathological anatomist, in their increase, and in the changes which they undergo. The instances adduced, however, are not very striking. Among others he cites that of the tumours of bones called *lardaceous*, in which, he says, the circulation no longer exists, but which nevertheless increase in size and alter in their texture, the hardest points at length becoming soft and fluctuating. The materials of the blood are effused in the parenchyma of the bone, but how could they penetrate thither otherwise than by imbibition? He thinks that these tumours live, like vegetables, by imbibition, and gives a case of a tumour occurring on the side of a girl's head, which M. Dupuytren attempted to remove, but found it impossible to do so on account of the connexion of the tumour with important parts. M. Magendie sawed off a portion of it weighing several pounds, and only a few drops of blood exuded from this large wound. He found that the tumour consisted of scirrhous and cerebriform matter deposited in the areolæ of the osseous texture, but there was no trace of sanguiferous vessels. The wound healed very speedily. The tumour grew again, and a portion was removed, the wound healing with uncommon readiness. Notwithstanding these partial reductions, the symptoms grew worse every day, and death impended. M. Magendie, therefore, ventured to tie the carotid artery, the result of which was that the tumour ceased to grow, and the patient's life was preserved. (Vol. i. pp. 119-20.) This was a bold piece of surgery, and, as it happened, successful as far as the tumour was concerned; we find afterwards, however, that the operation was followed by hemiplegia and permanent lesion of the mental faculties. (Vol. iii. p. 153.) We cannot perceive that this case affords any corroboration of our author's particular views. Many parts have blood-vessels which are too minute to be easily discerned, and we conceive that the tumour in question ceased to grow simply because its supply of blood was cut off. If it were otherwise, how should the ligature of the artery have had so great an effect? The tumour still retained its attachment to parts from which it might have imbibed nutrition.

As an illustration of the effects of dilution of the blood, M. Magendie gives the dissection of a dog, into whose veins he had injected, three days before, three pounds and a half of water, and which died two hours after the operation. According to the laws of imbibition, the liquid exhales by the readiest outlets. The lungs are most favorable to its elimination; hence a thick vapour proceeded from the throat of the animal, and as there was not time for all the liquid to be converted into vapour, a part of it was found under the form of a light froth. In this dog M. Magendie met with a phenomenon which he had not before observed: half an hour before death, the whole surface of the body was covered abundantly with a liquid, proceeding, as was supposed, from cutaneous evaporation. On dissection, the pelvis of the kidney is found to contain a small quantity of a serous fluid of a slightly red colour, the quantity of which would probably have been greater, but that imbibition into the neighbouring textures has taken place since the death of the animal. The intestines and other viscera are pale, and seem as if they had been macerated in water for a long time. The pleura is very moist, and appears charged with serosity, but there is no effusion of liquid within its cavity. The diaphragm has lost its natural rosy colour, and is studded with blue and livid spots, arising from the effusion of blood in the interstices of its fibres. The pulmonary tissue is gorged with liquids, and offers that first degree of alteration called *engouement.* The internal surface of the stomach is covered with the same spots as the diaphragm. (Vol. i. pp. 113-16.)

Commenting on the dissection of this dog, M. Magendie observes that the extravasation of blood into the cellular tissue is a frequent phenomenon in the human subject, in consequence of changes in the chemical composition of the fluids. One of the physical conditions of a normal state of the blood is its incapacity of transuding through the parietes of its vessels without the separation of its constituent parts; but if any modification of these constituents occur, as, for example, a diminution of viscosity by the injection of water into the veins, ecchymoses will appear in various parts. M. Magendie cites the instance of sea-scurvy, in which the blood, impoverished by the use of salt and scanty provisions, and the privation of fresh vegetables, is effused in large spots on the surface of the skin. In reference to the state of the lungs, he expresses his conviction that the modifications which the parenchyma of the lungs undergoes in pneumonia should not be referred exclusively to vital properties; there are also physical phenomena well worthy of attention.

M. Magendie remarks that electricity is not without influence on the phenomena of capillary attraction. Thus, if a capillary tube be immersed in a liquid, the liquid may be made to ascend rapidly into the tube by transmitting an electric current through it. This fact was first ascertained by M. Porret, and it was from the analogy of the results of his experiments and those of M. Dutrochet, that the latter gentleman was at first led to refer the phenomena of endosmosis and exosmosis to the influence of electrical currents, and to suppose that he had discovered the immediate cause of the vital movements.

M. Magendie relates an experiment of Fodera, showing the influence of electricity on imbibition. On placing some solution of prussiate of potash on the intestinal mucous membrane of a dog, and a solution of sulphate of iron on the corresponding serous surface, imbibition was at first very slow; but when a galvanic current was passed through the parietes of the intestine, the imbibition was much more rapid, and the surfaces were soon tinged with blue. (*Ib.* p. 118.) It results from these facts, says M. Magendie, that electricity may be usefully applied to therapeutics. He has himself applied it with success in the resolution of some tumours and obstructions. He observes that if the poles of the galvanic pile be applied merely to the surface of the skin, no remarkable result will probably be obtained, because electricity has a greater tendency to expend itself on the surface of bodies than to penetrate into their interior; he is, therefore, in the habit of driving two small metallic needles into the part he wishes to affect, and these being brought into contact with the pile serve as conductors of the galvanic current.* It is also known, says our author, that when a moist surface is subjected to the influence of electricity, evaporation goes on much more rapidly. (pp. 118-19.)

*Permeability of the Textures to Gases.* It is a point of great importance in physiology, observes M. Magendie, that all our textures are permeable to gases. A remarkable instance occurs in the lining membrane of the lungs; and here, also, we have imbibition by a double current, for while oxygen passes in to vivify the blood, carbonic acid passes out and mixes with the air about to be expired. The permeation of the textures by gases does not however take place on the same principle as the imbibition of liquids. The particles of liquids are united by cohesion, while those of gases, in virtue of their characteristic elasticity, tend to diffuse themselves through space. M. Magendie, therefore, believes that the penetration of gases into the pores of solids depends on their elasticity. He makes some observations on the effects of deleterious gases, and gives a remarkable instance of the apparent generation of fever by exposure to carburetted hydrogen. Some years ago a chamber, in which several persons were asleep, was suddenly filled with carburetted hydrogen proceeding from a gas-pipe. They were all immediately seized with typhus in a very severe form, which M. Magendie has no hesitation in attributing to the effect of the gas upon the blood with which it became mixed in the act of respiration. He ascribes to the agency of the same gas the morbific power of marsh miasmata. (p. 130.) But how does this accord with the known effects of carburetted hydrogen? When an atmosphere excessively charged with it is breathed for any length of time, it acts purely as a narcotic, inducing fatal coma; but it is well known that air, prettly largely impregnated with it, may be breathed with perfect impunity, as is daily done by miners and gas-men.† This being the case, we cannot help thinking that there must have been some other cause for the typhus mentioned by M. Magendie. Again, we cannot attribute the morbific effects of marsh miasmata to an agent which, though it doubtless exists in the atmosphere of swampy places, must be in a state of dilution, in which daily experience shows that it is innocuous, or produces at least no sensible bad effects. Neither do we see any reason to believe that an agent which produced typhus at one time would produce an ague or a malignant remittent at another: we can trace an affinity between the two latter, and can conceive them to originate from a common cause, varying in degree and modified by circumstances; but we can see no resemblance between either of them and typhus, nor any ground for ascribing them to a common origin with the latter disease.

M. Magendie lays great stress on the study of the effects of gases, and seems to anticipate great success from the use of remedies of the same

* This process was suggested by M. Berlioz in 1816, as a more efficacious modification of acupuncture, and put in practice by M. Sarlandiere, who published a work on *electro-puncture,* as he names it, in 1825. It does not appear generally to have met with much success.

† Christison on Poisons. 3d Ed. p. 743.

class, in cases where their deleterious action has been experienced. He gives the following illustrative experiment. An unstopped bottle of anhydrous prussic acid is passed rapidly under the nostrils of a small guinea-pig, and the animal falls motionless. The vapour of strong ammonia is then applied to the nose: the animal is agitated and utters cries; and M. Magendie has no doubt will survive the experiment. (Vol. i. p. 132.) He thinks that in cases of poisoning by prussic acid, immediate recourse should be had to the vapour of ammonia or chlorine; in like manner, if sulphuretted hydrogen were the deleterious agent, the first indication would be the introduction of chlorine gas into the lungs. M. Magendie is of opinion that the causes of many diseases reputed to be contagious might be found in changes in the atmosphere, produced by putrid effluvia—a point which we shall not discuss, as we have a dislike to reasoning without data.

*Viscosity of the Blood.* There is an important circumstance connected with the porosity of the animal textures, namely, that the orifices which give passage to the materials of nutrition are so small, that these materials can only enter the parenchyma of organs in a state of the most minute subdivision. Thus, while water, introduced into the stomach, is immediately imbibed, albumen, mucilage, or oil require to be reduced to very fine particles, by the process of digestion, before they can enter the lacteal vessels. It follows, as a necessary consequence, that if liquids be introduced into the blood, which are too viscid, or the particles of which are too large in proportion to the minute canals which they ought to pass through, the blood will stagnate in the vessels, and death will inevitably ensue, even though the substance introduced be perfectly innocent in its nature. (*Ib.* pp. 136-7.) These considerations, observes M. Magendie, suggest important cautions in the use of venous injections in the treatment of diseases. If, for example, a practitioner were to inject a solution of gum into the veins, he would immediately destroy his patient. In the direct introduction of foreign substances into the blood, it is of consequence also to be well acquainted with their chemical action on that fluid. Thus if corrosive sublimate were injected into the veins, or even an acid devoid of poisonous properties, the albumen of the blood would be coagulated, and the pulmonary vessels immediately obstructed. If mercury be injected into the veins, death follows by arrest of the circulation, and on dissection, a globule of mercury will be found obstructing each capillary vessel of the lungs. Why then can we administer it safely by friction or through the stomach? Because it is reduced to particles sufficiently minute to traverse the pores of the epidermis, or of the intestinal mucous membrane. A very curious phenomenon is observed when mercury is injected into the veins of a living animal: the lungs appear stuffed with an immense number of tubercles, and when these are examined, each puriform globule is found to contain at its centre a globule of mercury. It is, then, by the deposition of coagulated blood around these small foreign bodies that the numerous concretions are formed. (pp. 137-8.) M. Magendie asks if it be not possible that tubercles are formed in the lungs by a similar mechanism? The question is not new. Mercury has been injected into the lungs by M. Cruveilhier, Dr. Kay, and others with a view to this very point; but we cannot perceive that these experiments throw any light on the origin of tubercles.

Our author remarks that, when an animal is kept on too azotized a diet, symptoms occur which partly admit of explanation on the supposition of too viscid a state of the blood; and he is inclined to believe that in the diseases styled *carbuncular*, the abscesses formed depend partly on obstruction of the vessels from this cause. (p. 139.) He does not state his reasons for this belief, nor can we form the remotest idea of what they may be.

Some experiments follow, illustrative of the foregoing principles.

About a demi-gros (a drachm) of olive oil is injected into the jugular vein of a dog. The respiration becomes embarrassed, the thorax dilates with difficulty, and the inspiratory movements are multiplied to facilitate the passage of the blood through the capillaries of the lungs; life, however, does not appear to be endangered. Another quantity of oil, about equal to the former, is injected: the animal is agitated, and struggles, turns on his side, and dies suffocated. On dissection the lungs present every appearance of recent pneumonia, the crepitation is lost, and the density increased. When cut into they discharge a frothy and reddish serum. The blood is evidently more viscid than natural. The air-cells are gorged with a thick liquid, in the midst of which the oil may still be detected. The mucous membranes are pale. The crural artery contains only dark-coloured blood, which appears to be imbibed by the parietes of the vessel. This change in the colour of the blood is easily accounted for by the obstruction of the pulmonary circulation; but it is sometimes found where no such obstruction exists. M. Magendie has repeatedly observed that, in cases of apoplexy, the arterial blood, instead of being frothy and bright-red, is dull and blackish. He does not pretend to explain the cause of this; but he regards such a change in the colour of the blood as a most alarming symptom, and all the patients in whom he has observed it have died. (pp. 139-41.)

It is not only by the introduction of foreign substances that the blood becomes too viscid to circulate in the capillary vessels. Other causes lead to the same result. Thus, if, by too abundant a transpiration, a large quantity of serum be abstracted, the effect will be the same. The blood of an animal, the globules of which are large, will not suit the vessels of another animal with smaller globules. If, for example, the blood of a reptile were injected into the veins of a man, the want of relation between the fluids and the capillaries would inevitably cause death. (pp. 141-2.)

M. Magendie proceeds to make some remarks on the treatment of diseases, based on the principle that an impoverished state of the blood has a tendency to give rise to inflammation, and particularly to pneumonia. He states that he has ascertained this to be true, with respect to animals which have been debilitated by repeated abstractions of blood at short intervals. A case of tertian ague is given, in which a fatal degree of debility was induced by the injudicious and repeated employment of the lancet: on dissection, all the viscera were found healthy except the lungs, which were in a state of extreme *engouement*, strikingly analogous to that caused in animals by impoverishing their blood. M. Magendie believes that the secondary inflammations of the lungs and pleura, which supervene in the course of acute diseases, are often the result of excessive bloodletting. To this cause he also attributes those affections of the thoracic viscera in acute rheumatism which are usually considered as metastatic. He never employs bloodletting, either general or local, in this disease, and declares that in his own practice he never meets with these secondary affections. (pp. 144-8.) We cannot say that we think our author's view of the subject is borne out by the state of the blood generally observed in acute rheumatism, for of all diseases this is the one in which the blood has the greatest tendency to dense coagulation, often presenting a buff coat and a firm coagulum, even after large and repeated bleedings. We do not, however, mean to express ourselves advocates for large bleeding in this disease, as we are in the habit of using the lancet with great moderation, and that less as a direct remedy than as preparing the way for the more efficacious use of the colchicum. The fact stated by M. Magendie of the non-occurrence of secondary visceral inflammation, where bleeding has not been employed, is valuable as coming from so experienced a practitioner, whether his explanation of it be correct or not.

Returning to the subject of the viscidity of the blood, M. Magendie relates an experiment of M. Dupuy, in which water, holding in suspension fresh cerebral matter, being injected into the veins of a horse, caused immediate death, and on dissection directly after, the lungs were found gorged with blood, and their parenchyma filled with petechiæ. M. Dupuy saw these infiltrations of blood take place before his eyes. These results are attributed, both by M. Dupuy and M. Magendie, to the large size of the particles of nervous matter which prevents their transit through the capillary vessels. In these cases of artificial pneumonia, an injection cannot be made to penetrate the vessels which are blocked up with globules of the foreign matter. The arteries are almost empty and the veins gorged with black blood. (pp. 151-2.) A similar experiment is performed by M. Magendie on a dog, with a corresponding effect on the lungs; but the crural artery is found to contain florid blood, because the death of the animal has been so sudden that the arterial system has not had time to empty itself. (pp. 153-4.) From the rapidly fatal effect of the cerebral matter in these experiments, M. Magendie was at first led to suspect that it had some actual poisonous property; but he afterwards shows, by two other experiments, that this is not the case. The portal system, he observes, affords the best means of putting it to the test, since any substance blended with the blood of the vena portæ, must pass through the parenchyma of the liver, and undergo a complete comminution of its particles before it can pass into the general circulation. Having exposed a portion of intestine, he injects some of the cerebral emulsion, with Anel's syringe, into one of the branches of the vena portæ, having previously applied a ligature on the orifice in the vessel to prevent the escape of the liquid. No deleterious effect is produced. (pp. 159-60.) The other experiment also is very ingenious. It is well known, says M. Magendie, how rapidly the effect of a poison is developed by contact with the nervous pulp; if, therefore, we find that the substance in question, when injected directly towards the brain, produces not the effects of general poisoning, but simply those of obstructed cerebral circulation, we may infer that it is innocent in its nature. Some of the cerebral emulsion is therefore injected into the carotid artery of a dog. The animal immediately struggles violently, shows extreme anxiety, rolls himself up, and inclines his head forcibly to the side on which the liquid was injected; the eye of the same side is convulsively turned upwards, and agitated with a peculiar tremulous motion. Now, as these phenomena appear referrible rather to obstruction of the cerebral circulation than to any poisonous effect on the system, this experiment corroborates the conclusion from the preceding. The general inference seems fair enough, but our author is not correct in stating that the effects of poisons are very rapidly developed by contact with the nervous pulp, for it is well ascertained that poisons, which appear to act on the extremities of the nerves and through these organs on the brain and spinal cord, produce no effect whatever when applied to the cut surface of the brain and nerves, or to the latter in any part of their course.* This series of experiments concludes with one in which a gros (two drachms) of a concentrated solution of corrosive sublimate is injected into the jugular vein of a dog. The animal dies in the course of the day. On dissection the lungs are found hepatized and gorged with black blood. The right cavities of the heart are distended with fibrinous clots, and their lining membrane is blackened; the left cavities are collapsed and nearly empty. The usual effects of the poison on the intestinal mucous membrane are not observed, which M. Magendie attributes to the circumstance that, as digestion was going on at the time of the experiment, the chyle protected the intestine from the action of the poison.

These researches on the viscidity of the blood are of considerable interest both in a physiological and pathological point of view; and we shall have occasion to refer to the subject again, when noticing the contents of the fourth volume. We see no objection to the general mechanical principle which M. Magendie seeks to establish; he appears, however, to generalize too far when he predicts with confidence what would happen in man from what happens in a dog. Thus, although it is very probable that the injection of a solution of gum into the veins of a man would be fatal, we should not be in the least surprised if it were to turn out otherwise, because there may be a variety of circumstances of which we know nothing, in which the properties both of the blood and the vessels which contain it differ in animals of a different species; for example, how different must be the state of the capillary circulation and the relation of the extreme blood-vessels to the rest of the system in an animal which perspires, as man, and one that does not, as the dog.

*Elasticity.* Three kinds of elasticity, says M. Magendie, are admitted by natural philosophers: 1, the elasticity of *compression*, by which bodies yield to pressure, and resume their former shape when it is removed; 2, the elasticity of *traction*, by which bodies when forcibly stretched resume their original dimensions as soon as the force is removed; 3, the elasticity of *torsion*, by which bodies, when twisted, resume their original position when the force is removed. From elasticity also result those vibrations in the particles of bodies which give rise to different sounds. The organic textures are all endowed with elasticity, some of them in a very high degree; and, according to M. Magendie,

* Christison on Poisons. 3d Ed. p. 25.

many actions which have been attributed to peculiar vital endowments, depend entirely on this property. Various instances are adduced of elasticity in the different textures which we need not particularize, unless it be that of the elasticity of the lungs. M. Magendie maintains that it is to the loss of this property that the embarrassment of the respiration in cases of pulmonary emphysema is owing. He and M. Dupuy are convinced, from their joint investigations, that broken wind in horses is caused by a loss of elasticity in the lungs arising from emphysema. (pp. 169-70.) Our readers are aware that this is a very old opinion of pathologists.

The opinions of M. Magendie on the effects of the elasticity of the blood-vessels coincide with those of some other physiologists, who deny the influence of any vital contractility of these vessels on the circulation. There is nothing very new in the illustrations or reasoning adduced, and we shall not argue the question at present, because it will come before us more in detail in a future part of these lectures. The fact that it has been so much argued, and that the opinions of the best physiologists remain so much divided, is sufficient to show that data are still wanting for positive conclusions as to all the powers concerned in the circulation of the blood. Yet M. Magendie tosses to the wind all opinions opposed to his own as "a whimsical assemblage of absurd suppositions," and declares his belief "that the human mind never produced anything more ridiculous than the pretended mechanism by which it has been attempted to account for the capillary circulation." (pp. 185-6.) Is it not strange to find M. Magendie afterwards freely admitting that the various local changes which take place in the circulation, independently of the action of the heart, depend "on something peculiar—something which has not, hitherto at least, come within the domain of physics?" (Vol. i. p. 203.) These changes, he supposes, are produced by the influence of innervation; and he adduces the well-known fact, that when a part is deprived of its supply of nervous energy, the circulation is immediately disturbed, or even suspended. May not M. Magendie's "unknown something" consist in the vital properties of the extreme blood-vessels which he regards with so much contempt? And may not a cause which operates so energetically on some occasions, be constantly in operation to a certain extent?

M. Magendie makes some observations on the modes of arresting arterial hemorrhage, as acting on the principle that the three tunics of arteries are possessed of elasticity in different degrees. The operations noticed are the application of the *ligature—torsion—abruption,* or the tearing of the vessel—and *perplication,* a method proposed by M. Stirling, a German author, and which consists in making a small incision in the side of the artery near its bleeding orifice, then introducing a small pair of pincers, seizing the open extremity and drawing it backward through the aperture made in the side of the vessel, so as to form a kind of knot. The success of the three first of these operations is connected with the greater elasticity of the external tunic of an artery than of the middle or internal tunic; but we do not see what elasticity has to do with the effect of perplication.

The last hundred pages of the first volume are devoted to the consideration of *sounds,* normal and anormal, as produced in the human body, and particularly in the heart and arteries. With some glaring defects and more inconsistencies, this section contains much valuable and interesting matter. For the present, however, we pass it by, intending to return to its contents in an early number, when we come to notice some other works on the same subject.

In the volume which we have just closed, we have been startled with strange novelties, puzzled with conflicting experiments, and overwhelmed with a rapid succession of subjects, any one of which might afford diligent occupation for half a life-time. In the second volume, we are allowed more breathing time; its matter indeed is spun out, and all that it contains might have been set forth as lucidly in a third part of the space. After some general remarks, the greater part of which are a repetition of those made at the opening of the first volume, and some observations on various subjects too inconsecutive to require or admit of notice here, M. Magendie enters on the subject of hydraulics.

*Vital Hydraulics.* Our attention is here naturally directed, in the first place, to the heart, the great hydraulic machine by which motion is communicated to the mass of fluids. The investigation of its structure and action seems highly congenial to the spirit of our author's enquiry; an organ whose source of motion resides in an intense vitality, acts in a manner purely physical, and through the medium of a mechanical structure on the exact adaptation of which the life of the whole organism immediately depends, yet so intricate withal that the utmost ingenuity has hitherto failed to explain the precise use of all the parts which compose it. In the absence of any original observations on the subject, we think that M. Magendie should at least have given a somewhat elaborate and critical view of the opinions that have been advanced as to the moving powers of the heart. His observations, however, are extremely meager and commonplace, and the only novelty is an attempt to establish an analogy between the heart and a *pump*, in which he thinks himself so successful, that the two sides of the heart are thenceforth distinguished as the *right* and *left pump*. We submit, however, that there are two conditions of a pump which the heart lacks, and which, unfortunately for the analogy, are precisely those which constitute the essence of a pump—namely, the play of a piston and the formation of a vacuum. To speak of a pump destitute of these is about as correct as it would be to speak of a steam-engine without a boiler and without steam; we can therefore only regard the comparison between the heart and a pump as a mere conceit. That it is nothing better, is sufficiently evinced by the absurdity of the language to which it gives rise. Thus M. Magendie speaks of "the dilatation of the pump," (vol. ii. p. 106,) and tells us that "the liquid which comes from the reservoir is projected with a certain force against the walls of the cavity which receives it; this cause, entirely mechanical, contributes powerfully to the dilatation of the body of the pump." (p. 108.) We were not aware either that a pump dilated, or that it was in any degree indebted for its action to the fluid which it set in motion; and we are convinced that M. Magendie would have been the first to laugh at such phraseology as the above, had it been used by any one else. There are, indeed, but too many passages in these lectures which would afford a facetious critic abundant opportunity of indulging his vein; and it must be confessed that M. Magendie would have little right to complain if the shafts of ridicule were launched against him, for no one departs more frequently than himself from the grave and urbane deportment of the true philosopher. As one out of many examples, we may cite the following polite notice of the *eclectics*, or those who are fond of conciliating extreme opinions.

"If one could always discover and appropriate what is true in each system, this, without doubt, would be an excellent method. But do not trust these false illusions, these deceitful promises. Men should be judged of by their actions, not by their discourse. In what have these fervent followers of eclecticism deserved well of science? Do not the opinions they profess always betray their bastard origin? Poor eunuchs! Incapable of creating anything of themselves, they exhaust themselves in collecting here and there the fragments of errors which they pile up in the hope of arriving at the truth which they will never know." (Vol. ii. p. 67.)

We can assure M. Magendie that such rodomontade as this is neither conducive to the attainment of truth nor becoming those engaged in its pursuit. We must further maintain that, although the authors of original views have chiefly contributed to augment and impel the stream of human thought, the eclectics alone have directed its course in permanently useful channels, and led it to fertilize the fields of science. A teacher of any branch of knowledge, however endowed with creative genius, however distinguished for original discoveries, should always be an eclectic when in his chair; and placing his own opinions and those of others impartially before him, should separate facts from the inferences too frequently mistaken for them, and sound doctrines from specious theories. It is from the extreme difficulty of setting the mind free from an habitual bias, that many men, illustrious in science, make bad professors; their views require to be passed, with those of others, through the eclectic process, before they can become "profitable for doctrine."

To return from this digression—our author considers severally the functions of the two pumps, or, in ordinary language, the pulmonary and the general circulation.

*Pulmonary Circulation.* After some anatomical observations on the structure of the lungs, M. Magendie explains the pulmonary circulation in conformity with the opinion that the blood-vessels are simple elastic tubes—an opinion on which we shall have occasion to comment when we come to the general circulation. We meet with little otherwise which requires notice except some very peculiar pathological views, elicited by the presence of the influenza, which was very prevalent and fatal at Paris at the time these lectures were delivered. M. Magendie recurs to the experiments formerly rehearsed, showing that changes in the consistence of the blood cause effusion into the parenchyma of the lungs, disturbance of the circulation through them, and various changes of structure similar to those usually attributed to inflammation. There are some further experiments of a similar nature in the present volume, but they are scattered here and there with our author's characteristic want of arrangement. It were much to be wished that he had exhibited in a connected series all the experiments illustrative of the effects of foreign substances introduced into the blood, or the abstraction of any of its principles. We shall transfer hither some of those contained in this volume, and leave others until we come to notice the subject of the blood in the fourth volume.

A gros of sulphuric acid diluted with water is thrown into the jugular vein of a dog. The animal dies in a few seconds. The lungs are covered with brownish and livid spots; the right cavities of the heart are filled with clots, and the pulmonary artery and its branches are closed up by dense fibrinous masses; the left side of the heart is empty. Death seems here to be attributable to the sudden coagulation of the blood by the chemical action of the acid. (Vol. ii. pp. 179-80.)

A concentrated solution of subcarbonate of soda injected into the jugular vein of a strong dog causes instantaneous death. On dissection, the blood in the heart and large vessels and in the corpora cavernosa penis is found to be incoagulable; the lungs are in a state of *engouement*, and present the same brown spots and serous infiltration which M. Magendie has frequently observed in the lungs of animals which have died of hydrophobia: the cavity of the pleura contains a sanguinolent fluid; the liver, spleen, and kidneys, are softer than natural. (Vol. i. p. 319.) M. Magendie says he does not know whether any experiments have been made before his own on the introduction of subcarbonate of soda into the blood, and professes carelessness whether it be so or not, contending that the best plan is to make such experiments as suggest themselves, without reference to any which may have preceded them, and afterwards compare our results with those of others, so that what may have escaped one may be supplied by another. The only objection to this proceeding is that a man might spend his lifetime in ascertaining what was perfectly known before, or in drawing erroneous inferences from some source long since known to be fallacious. With regard to the effect of subcarbonate of soda on the blood, we may remark that this substance has been copiously introduced into the veins of the human subject in conjunction with the muriate, in the treatment of cholera by saline injections, without any bad result. M. Magendie afterwards finds that if water very slightly acidulated with sulphuric acid be added to blood, the coagulability of which has been destroyed by subcarbonate of soda, its coagulability is restored, and a clot formed exactly resembling the product of natural coagulation. The experiments were made on the blood of a guinea-pig. (Vol. iii. p. 75.)

We may add one more experiment illustrative of the effects of the *œnanthic ether*, a substance recently discovered by MM. Liebig and Pelouse, and which they suppose to be the principle on which the peculiar bouquet of wine depends. A gros of this liquor, mixed with an equal quantity of distilled water, is injected into the veins of a dog. The animal falls down intoxicated, and grows sleepy, the respiration becomes stertorous, and death takes place in three quarters of an hour. The principal appearances on dissection are engorgement of the lungs, a state of the intestines resembling inflammation, and other alterations, attributed by M. Magendie to the condition of the blood, which is found to be uncoagulated.

The following experiment is intended to illustrate the effects of abstracting a portion of the fibrine of the blood. Eight ounces of blood are taken from the jugular vein of a dog. The blood is agitated with a glass rod, and the fibrine is deposited in the form of yellowish filaments. The blood being then filtered through fine linen, is returned into the vein. The animal appears distressed, lies down, refuses food, and makes efforts to vomit. In the evening the same quantity of fibrine is again abstracted, and with similar but severer results; the animal becomes gradually weaker, the respiration is embarrassed, and death ensues. On the following day the body is remarkably rigid, somewhat swelled, and exhales a very fetid and putrid odour. On dissection the lungs are found hepatized; and there is a considerable quantity of reddish serum in the right cavity of the pleura. The heart contains a little serum with some small concretions suspended in it; but there is no true coagulation. (Vol. ii. pp. 196-200.) The experiment is afterwards repeated in a more gradual manner on several other dogs, a smaller quantity of fibrine being abstracted at once. In addition to the results already mentioned, inflammation and ulceration of the eyes and purulent discharges from the conjunctiva take place, and the skin is covered with vibices and petechiæ. (Vol. iii. pp. 324-9.) All these phenomena are attributed by our author to the changes in the blood induced by the abstraction of one of its elements. He draws no positive conclusion from the first experiment which he says came into his head only the day before it was made; but after repeated trials, he feels convinced that the effects produced were owing to the loss of the fibrine. We are amazed at M. Magendie's not being aware that experiments of the same nature were performed with very different results by Prevost and Dumas, some years ago; and that the results obtained by them have been fully confirmed by Dieffenbach* and Bischoff.† These enquirers have established beyond a doubt that blood deprived of its fibrine injected into the veins of an animal of the same species as that from which it was taken, or into the same animal, immediately restores it from the state of syncope occasioned by loss of blood, and that no bad consequences ensue. M. Magendie, indeed, afterwards alludes to the experiments of Dieffenbach and Bischoff, but merely states that those of the former *appeared* to succeed, while those of the latter failed entirely! (Vol. ii. p. 315.) The experiments of both these physiologists were perfectly successful and conclusive; and it is difficult to imagine the source whence M. Magendie can have derived such erroneous impressions with regard to them—surely not from the memoirs in which they are detailed by their authors. These experiments were instituted in relation to the subject of transfusion, and with a view to ascertain whether defibrinized blood could restore the actions of life; but if our object be simply to determine whether a change in the proportion of the fibrine to the other elements of the blood be productive of any immediate bad effects, there is no necessity for torturing unhappy dogs, because nature is continually making the experiment for us, and much more neatly, in the surgical wards of every hospital. When, for example, after the extirpation of a large tumour we find the cavity filled up in the course of a few hours with a dense mass of coagulated lymph, it is evident that a large quantity of fibrine has been suddenly abstracted from the circulating fluid, and that the blood must be minus this quantity. What ensues? Pneumonia, ophthalmia, and the putrid decomposition of the patient?—Not at all; the effusion of lymph is the sure forerunner of speedy reparation and recovery. We confess that we are quite puzzled to account for the

* Die Transfusion des Blutes. Berlin, 1828. † Müller's Archiv. 1835.

result of M. Magendie's experiments; but that there was some source of fallacy we have not the least doubt.

From the effects of alterations in the consistence of the blood, M. Magendie draws certain conclusions, which, if established, would render null and void at least one half of the science of pathology as it now stands. He objects to the term *inflammation*, as absurd and unmeaning, and seems to deny the existence of the state which it is intended to designate, attributing the phenomena usually so called to obstruction of the minute blood-vessels, owing to increased viscidity of the contained fluid, or extravasation into the parenchyma of organs from a preternatural tenuity of the blood. With respect to the term, it is singular that in almost all languages it involves the same idea, which looks as if there were something like common sense in its adoption. With respect to the state itself, we shall defer our observations till we notice the third volume, in which M. Magendie's views are more fully developed. The subject is only introduced at present in connexion with that of *influenza*. M. Magendie exhibits the lungs of numerous patients who have died of this affection. They present various degrees of engorgement and hepatization. These changes, according to M. Magendie, are the result of a dissolved state of the blood, which constitutes the essence of the disease. We cannot, however, see the slightest show of reason in favour of this opinion. That influenza is in many cases complicated with inflammation of the lungs is quite clear, both from the symptoms and from the benefit derived from the free use of the lancet. When such cases prove fatal, we believe that the inflammation, notwithstanding M. Magendie's contempt for that state, is abundantly sufficient to account for the appearances after death. We can conceive it possible, however, that in other cases similar changes may take place in the lungs, without any preceding inflammation; but here, instead of resorting with M. Magendie to a dissolved state of the blood, of the existence of which in the majority of cases we have not the smallest evidence, we should be inclined to regard an altered action of the vessels caused by disordered innervation as the source of the structural changes in question. The sufficiency of this cause for the production of the effect is well known, and is fully admitted and experimentally illustrated by M. Magendie himself. It appears to us, indeed, that the discovery of the influence of suspended innervation on vascular action, for which the world is indebted to Dr. Wilson Philip, has not been made sufficient use of in pathological reasoning; and we think it probable that many structural changes, which are attributed to inflammation, ought in reality to be referred to this cause. We are rather inclined to adopt this supposition, with respect to many of the local affections which occur in the course of adynamic fever, because disorder of the nervous functions is a prominent, and, perhaps, the most invariable and characteristic feature of this form of fever; because such disorder is known to be adequate to the production of the affections in question; and because these affections appear to differ from inflammation, both in their resistance to antiphlogistic treatment, and in the length of time they endure without inducing permanent change of structure. The same origin might, perhaps, be reasonably assigned to the organic changes in the viscera which are observed so frequently in the more protracted forms of insanity. But to return to the influenza: since this is evidently a disease affecting the whole nervous system, and the respiratory nerves in particular, would it not be more reasonable to ascribe the organic lesions which accompany it (supposing, with M. Magendie, that inflammation has no part in them,) to disordered function of these nerves, rather than to an alteration of the mass of the blood, especially as the changes are confined to the lungs?—whereas, if they arose from the state of the blood, they would be found wherever there was abundance of blood-vessels distributed in a lax cellular tissue. M. Magendie afterwards reads an extract from a letter he has received (p. 203), enquiring why, if the affection of the lungs in influenza depends on a change in the blood, all the vessels are not uniformly obstructed, and why one part of the lungs remains permeable, while others are hepatized? He acknowledges candidly that the objection is a grave one, and that he is unable to answer it. So much easier is it to frame short-sighted hypotheses than to answer the first and most obvious objections that may be raised against them!

It is singular that M. Magendie, in so often attributing local diseased actions to changes in the blood, forgets that an universal cause must have an universal effect, wherever circumstances admit of its operation; it is still more singular that he remembers this truth when it happens to suit his own purpose; for he adduces the fact that the *whole system* is affected in typhus in confirmation of his opinion that this disease consists essentially in a change in the *blood.*

"These congestions of the parotids, these pneumonic obstructions, these petechiæ, that putrid decomposition which so forcibly struck the old physicians—these are so many symptoms connected with a *general morbid principle.* What is the material agent destined to place in relation the different textures of the living economy? It is the *blood.* Suppose this liquid altered, and you will have an excellent reason for the disturbances which fall on each organ and each apparatus." (Vol. iii. p. 9.)

This reasoning, as far as relates to the general influence of the morbid cause (we speak not here of its nature) is strictly just; and if M. Magendie had kept it always in view, he would have seen that no local diseased action can be attributed exclusively to the state of the blood, although the effects of a morbid state of the blood may be made to manifest themselves in one part rather than another, by particular diseased conditions of the solids.

M. Magendie makes some observations on the morbid anatomy of the lungs, in conformity with his peculiar views. They contain nothing very remarkable, except an opinion as to the cause of pulmonary apoplexy, from which we must entirely dissent. "One thing," says M. Magendie, "is very positively known with respect to cases of pulmonary apoplexy, carefully observed—it is, that the blood is very thoroughly altered, and has lost the power of coagulating so as to form a firm clot." (Vol. ii. pp. 249-250.) He adds, "I have seen individuals struck with pulmonary apoplexy discharge blood by stool, by urine, by cutaneous transpiration, and by the principal exhaling surfaces." (*Ib.* p. 250.) Pulmonary apoplexy is doubtless sometimes accompanied with a general hemorrhagic disposition, as described by Laennec, Andral, and others; and such cases appear to be much more frequent in France than in England; but in the majority of instances there is nothing of the kind. Pulmonary, like cerebral, apoplexy occurs in connexion with the most opposite states of the vascular system and of the blood; and we should say that if any one thing be "very positively known" with respect to either, it is the frequent coincidence of disease of the heart, which M. Magendie might have pointed out as a cause quite as *physical* and much better approved by observation than an incoagulable state of the blood; the latter, indeed, must be excluded even as a possible cause where the disease occurs, as it often does, in persons labouring under active plethora and too dense a crasis of the blood.

The latter part of the second volume and the whole of the third are occupied with the consideration of the circulation.

*General Circulation.* Our author's opinions on this subject are based on the supposition that the heart is the sole source of the power by which the blood is propelled throughout the system; the arteries, large and small, as well as the capillaries, he regards as simple elastic tubes, which, being distended by the blood, contract on it in virtue of their elasticity, and render continued and uniform the successive impulses it receives from the heart. That the larger arteries are not possessed of any power which can properly be called muscular, and that their ordinary action on the blood consists principally in an uniform contraction, which propagates an impulse originally received from the heart, is, we think, sufficiently established; that they possess elasticity is unquestionable; but that their contractile power resides solely in this property, we utterly deny, and we do so on *physical grounds.* 1. It was distinctly proved by the experiments of Hunter and Parry—and the fact may be verified by any one who will take the trouble—that the arteries contract, immediately after death, to a caliber smaller than that which they maintain some time after death, but before putrefaction has commenced: now the time to estimate the effect of mere elasticity in an artery, is evidently in the interval between the entire extinction of vitality and the commencement of decomposition, for the vessel is then possessed of its simple physical properties, and no other. 2. M. Poiseuille has demonstrated that if the artery of an animal just killed be distended with water, the force with which it contracts, in expelling the water, is greater than the force used to distend it, and greater than the same artery exerts a short time after death. 3. An artery of moderate size, if divided transversely during life, will contract to a point; that is, to a diameter smaller than that which it preserves after death.

From these facts, two conclusions appear inevitable; first, that the contractile power of the arteries depends on something more than mere elasticity, since it produces effects to which elasticity is unequal: secondly, that this power is a vital one, since it ceases with life. The hypothesis which regards the arteries as simple elastic tubes, though essentially false, does not lead to any very important practical error, so long as its application is confined to the large arteries, because, although the proper contractile force of these vessels differs from elasticity, both in kind and degree, it has nevertheless an analogous influence on the course of the blood; namely, that of continuing the impulse of the heart by an uniform contraction. When, however, we approach the minute extremities of the arterial system, the case becomes widely different, since we are here brought in contact with a new set of phenomena, and have to consider, not merely the force which propels the blood, but the means by which its movements are so regulated as to render it available for the purposes of life. It is only very lately, that the enquiry concerning the influence which the capillaries may exert on the circulation, by their vital actions, has been properly limited ; indeed it still continues to be very vaguely pursued by many physiological writers. The question whether these vessels do, by their vital contractions, add directly to the force which *propels* the blood, is perfectly distinct from the question whether they do, by vital actions of some kind, regulate the *distribution* of the blood. The first question, we conceive, must be answered in the negative; because, admitting them to possess a vital power of contraction, the exercise of this power would antagonize the action of the heart, instead of aiding it. The second question is more difficult of solution ; but as it is one of infinite importance to physiology, we will dwell on it for a moment. If we consider the capillaries as endowed with a vital power of diminishing their tube, and thereby lessening the quantity and augmenting the velocity of the blood which passes through them, and of dilating and thereby increasing the quantity and diminishing the velocity, we must admit that these vessels, by their vital actions, contribute most powerfully to the circulation of the blood, which, in the true physiological sense, does not mean the mere performance of a course from the heart, through certain tubes and back again, but the distribution of the vivifying fluid to every part of the frame, in a manner suited to the maintenance of nutrition, secretion, calorification, and other functions on which life intimately depends. Do the capillaries, then, possess such powers? We think there is evidence, little short of demonstration, that they do. As it is admitted on all hands, that local changes of diameter take place in the capillaries, independently of the action of the heart, what we have to ascertain is, whether such changes depend on mechanical or vital causes. Now many of these changes evidently take place under the influence of innervation. The glowing countenance of anger or shame, the alternately flushed or pallid visage of agitation or embarrassment, are universally referred to the influence of the nerves, the only channels through which the mind conveys its impulses to the corporeal organs. This is fully admitted by M. Magendie, who instances also the effect of dividing the pneumogastric nerve on the pulmonary circulation, than which no instance can be more to the purpose. If, then, sudden changes in the innervation of a part, have so marked an effect on its vascular functions, it cannot reasonably be denied that the ordinary and continued influence of the nerves is, in all probability, an important condition to the maintenance of those functions. But how do the changes of innervation affect the diameter of the vessels? It can only be by modifying their elasticity, or by influencing their vital properties. Now what ground have we for supposing that the nervous influence can effect an immediate change in the elasticity, or any other physical property of a texture? None whatever; there is no fact in physiology which affords the shadow of support to such an opinion. But we have ample proof that the nervous influence is capable of producing an immediate action, by affecting the vital properties of a texture, as when it causes the contraction of the muscular fibre. The influence of innervation, then, must be exerted, not on the elasticity but on the vital properties of the blood-vessels. It is true that some physiologists, and among them the distinguished Müller, have recently attempted to explain the phenomena in question, by the play of certain *vital affinities.* They suppose, that the influence of the nerves is to increase or diminish the vital affinity between the blood and the textures in which a change of distribution takes place; but, with deference to high authority, we must submit that the expression *vital affinity* appears to us exceedingly vague and indefinite, nor can we help suspecting that those who employ it would, if pushed hard, find considerable difficulty in explaining what they mean by it. Moreover, admitting the existence of such an affinity, we do not perceive how it could increase the quantity of blood in a part, unless new vessels were created, or those already existing dilated for its reception. The former supposition, we presume, will not be maintained, nor does the latter appear more tenable; for how can an *affinity,* in any conceivable sense of the term, dilate a vessel by forcing blood into it, in opposition to its mechanical tendency to contract? We must, therefore, conclude that the nerves do, in all probability, influence the vital properties of the capillaries, and thereby occasion changes in their diameter which regulate the distribution of the blood, and hence act a very important part in the circulation.

We have purposely abstained from enquiring whether the capillaries possess the texture of arteries and veins, or form a distinct order of vessels. We have made no attempt to determine whether the small arteries, which lead to the capillaries, possess vital endowments, similar to those we have attributed to the latter. These, and other disputed points, were not essential to our object, which has been to point out a few phenomena, the existence of which cannot be disputed, and to show that these phenomena necessarily involve the presence of a vital power in the extreme blood-vessels, which influences the distribution of the contained fluid. We are thus enabled to place in a broad light the fallacy of the doctrine, that the arteries are mere elastic tubes—a doctrine which, if admitted, would go far to divert all attention from enquiries which, in the present state of physiology, are perhaps of greater importance than any other; namely, those researches into the arcana of the capillary system, for the success of which the science is waiting, as for the probable means of indefinite extension. These things being premised, we are prepared to follow M. Magendie in his observations on the action of the blood-vessels, considered simply as parts of an hydraulic apparatus. He describes the instrument invented by M. Poiseuille, for estimating the force with which the blood moves, a very ingenious contrivance, which promises to be of great utility in researches of this nature. It is simple, of easy application, and seems to afford very uniform and accurate results.* M. Magendie repeats Poiseuille's experiment, showing that the force of the blood's motion in all arteries is the same, whether the vessel be situated near the heart or at a distance from it. Hence, if we ascertain

* A figure of it, with explanations, will be found in the Cyclopædia of Anatomy and Physiology, and in Dr. Baly's translation of Müller's Elements. The worst thing about this instrument is its name—*hemodynamometer;* this, although more than sufficiently sesquipedalic, is yet too short for good etymology, which would make it *hematodynameometer*. See a good paper on this instrument, as indicating "the physiological effects of various agents introduced into the circulation," by Mr. James Blake, in the Edin. Med. and Surg. Journal, for January, 1839.

the degree of pressure of the blood in any one artery, by the height of the column of mercury which it will support, the amount of pressure in any other artery of a given caliber may be ascertained, by multiplying the area formed by the circumference of the vessel by the height of the column of mercury which is supported by the pressure of the blood, as already ascertained, in the first artery. M. Poiseuille found, also, that the pressure of the blood was the same, whether the animal experimented on were large or small. On this M. Magendie remarks, that there is a circumstance which may influence, in some degree, the accuracy of the results obtained by M. Poiseuille's instrument: a certain quantity of blood mixes with some subcarbonate of soda, which is put into the tube in order to maintain the fluidity of the blood. In large animals the loss of this quantity of blood is insignificant, but in small ones it should be taken into account. (Vol. iii. p. 45.)

By the use of the same instrument it is demonstrated that the force of the blood's motion is remarkably increased by expiration and diminished by inspiration, a fact previously observed both by Haller and Magendie, and which depends on the increase or diminution of pressure on the large vessels by the contraction or dilatation of the thorax. M. Magendie affirms that the influence of the respiratory movements is much less in the crural than in the carotid artery, because, by the descent of the diaphragm during inspiration, the abdominal viscera are made to press upon the blood-vessels, and during expiration this pressure is removed, so that the effect of the movements of the thorax are in some degree antagonized by those of the abdomen. This differs somewhat from the observations of Poiseuille, who states that in ordinary respiration the increase or diminution of pressure is the same in all the arteries, but that during violent respiratory efforts the effects are much greater in the arteries near the heart than in those at a distance from it.*

M. Magendie makes an experiment to ascertain the effect of increasing the volume of the circulating fluid, which, he anticipates, will be to augment the pressure of the blood. He injects some tepid water into the jugular vein of a dog, but, contrary to expectation, the instrument indicates a diminution of pressure. (Vol. iii. pp. 50-1.) On reflection, he attributes this to the debilitating effect of the water on the muscular fibres of the heart. The experiment, however, being repeated, with transfused blood instead of water, no change of pressure is indicated. In a subsequent trial blood being withdrawn, by means of a syringe, from the crural artery of a dog, and the same blood being reinjected, the instrument marks a diminution of pressure as the blood is withdrawn, and an increase of it as it is restored ; this, however, may be merely on the principle that the action of the heart is depressed by withdrawing its natural stimulus, and raised again by renewing it. (pp. 124-5.) An attempt to make a similar experiment on the jugular vein is frustrated by the sudden death of the animal from the introduction of air, and also, according to M. Magendie, from the sudden coagulation of the reinjected blood. (pp. 125-7.) He therefore, very properly, pronounces all these experiments inconclusive. We think, further, that it would be extremely difficult to devise any conclusive experiments on this subject, because

* Magendie, Journal de Physiol. Tom. viii. p. 296.

the increased volume of fluid might operate not only by its mass, mechanically considered, but by influencing the irritability of the heart, and most probably influencing it differently at different times, according to the vital condition of the organ. An aqueous infusion of coffee, injected into the veins, causes a marked increase of pressure by augmenting the action of the heart. Diluted alcohol has no effect. (pp. 52-4.)

While the pressure of the blood is equal throughout the arteries, it varies in every part, and almost in every tube, of the *venous system.* By the application of Poiseuille's instrument, M. Magendie demonstrates it to be much greater in the crural than in the jugular vein. The slower movement of the blood in the veins than in the arteries seems to depend on the greater capacity of the former vessels, rather than on the diminished influence of the propelling force of the heart; for M. Poiseuille has found that if all the blood carried to a part by an artery is made to return by a single vein, the pressure in the two vessels is nearly equal. M. Magendie repeats an experiment of Poiseuille's, illustrative of this fact. The crural artery and vein of a dog being insulated from the surrounding textures, the circulation through the other vessels of the limb is suspended by two tight ligatures: the pressure in the vein becomes equal to that in the artery. Even before the ligatures are tightened, the pressure in this vein is found to be very considerable, because the other veins are but small, and a great part of the blood returns by the crural vein. (Vol. iii. pp. 181-3.) On the preceding principle, the pressure in the deep veins is much greater than in the superficial, because the latter are much more numerous; and hence, observes M. Magendie, the much greater difficulty of suppressing hemorrhage from deep than from superficial veins.

Our author commences his investigation of the capillary system in a manner very characteristic of him. In alluding to the opinions generally maintained, he remarks ironically, that "there is nothing but extraordinary phenomena, from the sensibility which is insensible to the contractility which it has not been vouchsafed to any one to see. A globule of blood wishes to move in a particular direction—for it also has a will of its own;—the capillary will not let it. The vital laws are at strife with the physical; happily the former are victorious." This is smartly said, and if Verschuir, Thomson, Parry, Hastings, and many others, profess actually to have seen the contraction of the capillaries on the application of stimuli, who are they? *Nobody!* All of them put together would not make a physiologist of decent dimensions, according to the conceited estimate of M. Magendie. We shall not follow M. Magendie throughout his observations on the healthy functions of the capillaries; for there is nothing new in them, except the dogmatical decision of questions which the best-informed physiologists approach with diffidence, and regard as quite undetermined. We have already stated our acquiescence in the opinion that the capillaries do not contribute by their vital contraction to the propulsion of the blood, but we have also stated the grounds of our conviction, that the minute arteries, in the exercise of their vital powers of contraction and dilatation, afford indispensable aid to the circulation, by regulating the distribution of the blood and the various processes immediately dependent on it.

M. Magendie gives a summary of the observations of M. Poiseuille on the manner in which the blood traverses the vessels. All the observations of so ingenious and accurate an enquirer are worthy of attention, and we recapitulate these, as they have not yet found their way into the treatises on physiology. When a liquid passes through an inert tube, a portion of it adheres to the parietes of the tube, as by a species of attraction, and remains motionless. M. Poiseuille has found that this holds good, also, with respect to the passage of the blood through its vessels; an immovable layer of serum rests in contact with their parietes, while the blood traverses their tube. If the circulation be examined in a vessel large enough to admit of the passage of several globules abreast, their motion is perceived to be very rapid in the axis of the vessel, and less so near the parietes; while in the layer of serum adhering to the latter, the globules do not move at all. In the axis they have only a progressive movement, near the layer of serum they have a progressive and a rotatory motion; the latter being more conspicuous the nearer they approach it: the globules which are entangled in the substance of the serous layer become immovable, and remain so; those which merely touch it, roll on their axis as if they impinged on an undulating surface. The motion of the globules is slower in the distal portion of an artery than in that nearer the heart. The irregularities in the movements of the globules are referrible to their position in relation to the adherent layer. Thus, two globules may be moving abreast with equal velocity; one of them, jostled by its companion, is pushed towards the circumference of the vessel, and its progress is retarded; a shock from a new globule restores it to the centre, and it recovers its former velocity: at another time, a globule gets placed crosswise of the vessel, and its extremities become immersed in the immovable layer, its progress is therefore retarded; but others pressing upon it as it intercepts the passage, it soon yields, becomes longitudinal, and resumes its course along with the rest. These accumulations of globules happen very rarely, when the heart retains its full power; they are hence generally observed only towards the close of an experiment, when the animal is weakened. In the great vessels, the globules moving in the axis are not at all influenced by the immovable layer at the circumference, on account of their distance from it; in the capillaries, on the contrary, they are all obliged to traverse a mass of serum, and the central current only has a certain degree of velocity. (Vol. iii. pp. 259-61.)

Although we honestly confess that we are somewhat tired of wading through the deluge of ill-ascertained facts, hasty deductions, and gratuitous hypotheses, with which this work is overlaid, we must notice M. Magendie's views on the subject of *inflammation*, before we dismiss the third volume. These views have been several times partially introduced, and always with much confidence and infinite contempt of existing opinions: we might, therefore, naturally have expected a very distinct statement of them, and very striking facts, luminous illustrations, and conclusive arguments in their support. We acknowledge, however, that we have been more afflicted than surprised to find that our author ends in having no settled opinion on the subject. We have already seen that M. Magendie attributes what is called inflammation to mechanical obstruction of the capillaries from increased viscidity of the blood, or to extravasation of some of the elements of the blood into the texture of the part affected, owing to a morbid tenuity of that fluid. In entering more particularly into the subject, he attempts to explain the origin of inflammation as follows. If an alkali, or other chemical reagent be applied on any point of the mesentery of a living animal, the circulation is immediately arrested at that point. A dark immovable spot is perceived, all around which the capillaries are turgid, because the blood which should have flowed through the obliterated vessels, passes into the neighbouring ones, and distends them by its pressure. In this instance, the chemical agent causes a *racornissement* or crispation of the parietes of the vessel; but similar consequences ensue, whenever the normal relation between the diameter of the capillaries and the volume of the contained globules is subverted. (Vol. iii. pp. 423-4.) This hypothesis differs little from that of Boerhaave; its essence is obstruction—the globule being too large for the vessel, or the vessel too small for the globule. According to M. Magendie, the principal phenomena of inflammation are easily accounted for on these data.

"The analysis of the course of the blood, in a part struck with inflammation, explains the signification of the four famous words *dolor*, *calor*, *tumor*, *rubor*. The pain results from the compression of the nervous filaments by the obliterated or distended vessels; the heat indicates, that the blood passes in more voluminous columns in the neighbouring capillaries which remain pervious; the swelling proceeds from the dilatation of the vascular net-work, and the extravasation of the materials of the blood; the redness depends on the presence of an increased number of globules in the vessels." (Vol. iii. pp. 128-9.)

M. Magendie regards the phraseology which expresses the dependence of inflammation on irritation, as a mere mystification of the old axiom, *ubi stimulus ibi fluxus*. If a puncture be made in the mesentery of a living animal, the blood flows from all quarters towards the injured point, and what is called inflammation is set up. This, say the physiologists, arises from *irritation;* but M. Magendie assures us that it is a mere hydraulic phenomenon: a capillary is punctured, the blood runs out, and, by a necessary consequence, that contained in the neighbouring vessels is determined towards the aperture, whatever were its previous course. He has observed, that if the tissue of the membrane merely be punctured, the course of the blood undergoes no change; he asks, therefore, how it can happen, if the phenomena depend on irritation, that the stimulus produces the effect when applied to the vessel, but not when applied to the membrane, the sensibility of the latter being infinitely the greater? We need scarcely suggest, that he is here confounding *animal* with *organic* sensibility; but setting this aside, we do not perceive how a visible change can take place in the course of the blood, where no blood-vessel is visible. The most familiar of all instances sufficiently shows the futility of M. Magendie's hypothesis. If the slenderest hair, or an impalpable particle of dust, get on the surface of the eyeball, in a person in whom the organ is at all irritable, the vessels of the conjunctiva immediately become injected in all directions; *ubi stimulus ibi fluxus:* but no capillary is punctured in this case; the *irritability* of the vessels —that despised and proscribed property—is alone concerned in the phenomenon. We shall not discuss the merits of M. Magendie's theory of inflammation, because, in so doing, we should merely be enquiring whether the pathology of that state had been advancing or retrograding for the last century; and were it otherwise, it would be scarcely worth while to contest opinions which the author himself fairly leaves in the lurch. He begins the last of these lectures with the following announcement,—a remarkable one, certainly, after the unhesitating confidence with which he has previously introduced his own views, and pronounced those of others to be nonsense:

"It was my intention to have delivered, at the end of this course, the complete history of inflammation; but it has fared with me as with a traveller who views an object from afar. Distance, at first, prevents its true character from being appreciated: it appears small. As it is approached, its proportions increase; yet nearer, they expand still more, and finally, when we come close to it we are frightened at its gigantic size. Accordingly, I thought that a few meetings would suffice to consider all the aspects and explore all the depths of the important phenomenon of inflammation. I was mistaken. This question is much more complicated than it appeared to me at first sight, and for its study it is not sufficient to contemplate it at a solitary point of the capillary system. We must take into consideration each organ, and each texture, for they have each their individual mode of circulation; we must examine their vascular texture, and the causes which modify the course of the blood in their parenchyma, and thus rise gradually from exact physiological knowledge to the experimental analysis of inflammatory disorders." (Vol. iii. pp. 440-1.)

Entirely acquiescing in these sentiments, we may venture to suggest that candour in acknowledging hasty or erroneous opinions, though always laudable, might in many cases be advantageously superseded by the caution which would prevent their adoption.

In entering upon the fourth volume of these lectures, which is entirely devoted to the consideration of The Blood, we must refer our readers to an article on the same subject in our twelfth Number, (*Br. and For. Med. Rev.*, vol. VI. p. 431,) to which we wish the following remarks to be considered as supplementary. In that article, we asserted that but little had as yet been done to aid our knowledge of the pathological changes which the blood undergoes in different states of disease. From the reputation of M. Magendie, we expected to find in these lectures, that this great deficiency would have become at least in part supplied by his experimental researches. But we regret to say that we have been as much disappointed in this expectation, as we have been in so many others, raised by the previous reputation of their author. No portion of the present work presents a more remarkable illustration of our Bacon's "Idola spêcus" than these lectures on the blood; and we think we may venture to affirm that saying this is equivalent to saying that no better illustraton is to be found in the whole course of modern medical literature.

The greater part of this volume is occupied with an examination of the properties of fibrine, the phenomena attending the coagulation of the blood, and the physical circumstances under which it may be retarded or accelerated. The properties of albumen and colouring matter are more briefly discussed, and the saline and other substances contained in the blood are scarcely noticed. The first lectures are occupied with a general sketch of the objects of the course: the author avows himself to be an ultra-*humoral* pathologist, conceiving that the sources of most diseases must be referred to changes in the blood. These changes he is for the most part disposed to regard as purely physical. Two conditions he considers to be indispensably necessary for the maintenance of life and health: 1, That the blood should consist of a certain proportion of liquid and solid parts; and, 2, That it should be in continual circulation throughout the system. The discursive style in which the lectures are given renders it rather difficult for us to condense the author's views.

"The blood," he observes, "in the living animal, easily circulates through the minutest capillaries, but if any one of its properties be altered, it has no longer the power of traversing those vessels; it becomes effused in the surrounding tissues, giving rise to extravasation, œdema, and inflammation. . . . The following experiment may be adduced in support of this opinion. This vessel contains liquid blood: it has the normal proportions of serum, globules, and saline matter. In short, it contains twenty-four out of the twenty-five principles which have been announced by M. Lecanu to exist in human blood. One principle is wanting, but the deficiency is not appreciable to the eye, and the blood appears in every respect analogous to that which circulates in the living animal. Nevertheless, if reintroduced into a vein, it will at first traverse the larger trunks, but when it reaches the capillaries, its course will be arrested; it will become extravasated, and the animal will soon die from this stoppage of the circulation in the capillary system. Yet nothing has been added to this blood. We have simply abstracted from it one of its principles which forms not more than one or two thousandths of the mass. I allude to fibrine, a substance which is liquid in the vessels, but which becomes solidified so soon as it has escaped from them. Hence it appears it is the fibrine which confers on the blood the property of traversing the most delicate capillary vessels." (Vol. iv. p. 43.)

We cannot agree to this conclusion, which seems to us to be little warranted by the experiment. Had the author removed the colouring matter or the albumen, or the saline matter only, and left the other principles, we are pretty well assured that the capillary circulation would have been equally disturbed, and would have led to serious consequences. At any rate, until experiments had been performed on the other principles individually and with negative results, it is not justifiable to ascribe to the presence of fibrine alone the property which the blood has, of traversing the capillary vessels. He seems to have felt in some degree the weakness of his position, for immediately afterwards he says:

"The blood may contain fibrine and its other constituent parts, but if a substance capable of chemically combining with it and forming a salt, as potash, soda, or ammonia, be injected into the vessels, the fibrine loses the power of coagulating; the blood not containing this principle in its *normal* state, will itself cease to be coagulable, and consequently will not have the power of circulating freely through the capillaries. Thus even fibrinous blood may be unfitted for circulation. Here then we have a valuable and fundamental fact in the history of the blood, namely, that in order to support life, this liquid must have the property of coagulating. When this is not the case, death is a speedy consequence." (*Ib.* p. 44.)

"The causes which deprive fibrine of its property of coagulating exist in the air, in miasmata, in food, in short in all that surrounds us, and becomes in any way a part of our organization. Connected with these researches, we may notice the employment of certain medicines calculated to give rise to serious consequences. Thus the use of carbonate of soda carried to a great extent, as it sometimes is in calculous disorders, may become injurious. The explanation is as follows: this substance has the property of rendering the blood liquid by combining with the fibrine. I am *persuaded* that from the too frequent use of this salt, the blood becomes changed,—liquefied,—hence there are infiltrations in the lungs, followed by successive attacks of pneumonia.

This at least occurred in the case of one of my friends, who was obliged to give up the use of the medicine." (Vol. iv. p. 48.)

Here we see the following points assumed: 1, That carbonate of soda, introduced into the stomach, operates chemically on the blood, in the same way as when it is injected into a vein; 2, That in both cases it acts only by liquefying the fibrine, and consequently obstructing the capillary circulation through the lungs; 3, That miasmata or febrile poisons and alkaline substances affect this liquid in a similar way, and cause death by giving rise to similar changes in its properties. The physical alteration in the blood is all that M. Magendie appears to look to; and whether it be due to carbonate of soda injected into a vein, or to the contagious miasmata of fever, is a matter apparently of little importance. The fibrine having lost its coagulating power, the blood, it is alleged, cannot circulate through the capillary vessels, and hence death ensues. On this principle, he says, artificial diseases may be formed "*de toutes pièces,*" (p. 7;) and, at the pleasure of the experimentalist, animals may be made to suffer from pneumonia, scurvy, yellow fever, typhus fever, &c. (p. 5.)

Admitting that carbonate of soda taken internally is actually absorbed as such into the blood, a point which M. Magendie assumes, we have yet to learn whether, when the salt is in that liquid, it operates only by annihilating a property of fibrine which does not belong to that principle until after its removal from the living vessels. Fibrine is already liquid in the blood,—how therefore can it be said that the salt acts by *liquefying* what is already liquid? But let us advance a step further and admit that the alkaline substance destroys a property not possessed by fibrine in the living body, it has yet to be proved that the liquid compound of fibrine and soda acts fatally in obstructing the capillary circulation. Is not the salt as likely to be mixed with the albumen as with the fibrine, and might it not act fatally on the system, when thus mixed up with the albumen or serum? M. Magendie supposes that it combines with the fibrine alone, when introduced into the blood; and that it thus destroys life: but we search in vain throughout the volume for a single experiment to countenance this supposition. In short, all we learn from his statements is that a salt which retards or prevents coagulation in blood removed from the body may destroy life when introduced into the circulation. The *modus operandi* is left wholly unaccounted for. Some salts have but little effect on the coagulation of blood; others accelerate it; but the author has not deemed it necessary to perform parallel experiments with these: and yet it is clear, if these salts, when injected, produce the same degree of mischief in the animal, his hypothesis must fall to the ground. Can an animal bear with impunity the injection of a saturated solution of alum into its blood? We think not. Alum, according to most chemists, has the property of favouring the coagulation of that liquid when removed from the body; and yet it remains to be proved whether it will not destroy life as readily as carbonate of soda, although the latter has the reverse action on blood. In truth, it appears to us wholly unjustifiable, in the present state of our knowledge, to assert that the action of salts introduced into the circulating fluid is at all indicated by their power of preventing or favouring coagulation. We say nothing of

Magendie's view of the supposed serious consequences liable to follow from the medicinal use of the carbonate of soda, since experience plainly shows that he is in error. Most practitioners must have seen this salt taken in considerable quantities by healthy individuals, and for a long period together, without any such effects ensuing as those described by him. (p. 41.) Again, because the blood does not coagulate when drawn in certain diseases, as cholera and yellow fever, miasmata are supposed to operate like carbonate of soda in keeping liquid, the fibrine. This speculation is ingenious, but we do not see, even if it be admitted, how it advances our knowledge of the causes of non-coagulation in these diseases, or of the cause of death.

After denouncing the modern doctrines of inflammation and irritation as romantic fables unworthy of science, the author proceeds to remark on the *viscidity* of the blood, as a condition indispensable for its circulation through the capillaries. He once more refers to the interesting observations of M. Poiseuille already noticed, which prove that water cannot be forced through tubes of small caliber, even with the greatest pressure; but that if a certain portion of mucilaginous matter be added to the water, such as gum, gelatine, or albumen, then the injection is easily accomplished. (p. 57.) M. Magendie assures us that if the viscidity of the blood be diminished, by the abstraction of successive portions and the substitution of water, the circulation is impeded, and the extravasation with serous exhalation follows; if it be rendered more viscid than natural by the introduction of any foreign substance, then its course is arrested through the close adhesion between it and the parietes of the vessels.

We find nothing of importance on the subject of the coagulation of the blood; but the author admits the liquid to be of a very different nature in and out of the body. In relation to the proportions of serum and crassamentum, he considers that where the serum is unusually abundant, venesection ought to be avoided, and this he believes to be an opinion which sooner or later will be recognized as a fundamental doctrine in the treatment of disease. An experiment performed on a dog, however, showed that, although this animal had been three times bled, and distilled water substituted each time for the blood abstracted, the result did not bear out this view. The blood, when drawn for observation, yielded but little serum. In relation to this unsatisfactory result, the author very properly observes that one fact cannot overthrow another. (p. 105.) This we freely admit; but at the same time, when such apparently conflicting results present themselves, we are not in a condition to draw a conclusion one way or the other.

The detection of albumen in the urine has been in general considered to indicate the existence of disease of the kidney: in our last Number we showed that this was a mistake: and M. Magendie informs us that he has found that a superabundance of serum in the blood is attended with albuminous urine. Thus human serum was injected into the veins of a dog: on examining the urine of the animal, and, at the same time, a specimen of urine from a patient labouring under diseased kidney, albumen was discovered in each, in apparently the same proportion. After death, the body of the dog presented no trace of diseased kidney. (p. 148.)

The author considers it a matter of extreme importance to trace out those substances which have the property of liquefying the blood, in other words of preventing its coagulation out of the body. One of these, the carbonate of soda, has already been alluded to. Putrid water is another agent of this description; when injected, it destroys life by rendering the blood liquid, but the chief seat of its action is on the intestinal canal. (p. 199.) The various kinds of fever may be considered as having their origin in miasmata introduced into the lungs, and through those organs into the blood. In the subjects of these diseases, he believes the blood to be altered in its properties, but has not been able to verify this by observation.

The effects of certain reagents on the blood are next adverted to. *Sulphuric acid* is generally set down as accelerating coagulation, and thus it has been used internally and externally as a styptic. The author from one experiment comes to a directly opposite conclusion. Sulphuric acid mixed with blood, diluted with an equal part of water, merely rendered the colouring matter black: there was no solidification of the fibrine. Hence it is inferred that this acid, administered internally, instead of favouring coagulation, may actually prevent its occurrence. (p. 204.) The result of this experiment might be accounted for by the very small quantity of sulphuric acid used; but whatever explanation we may adopt, we cannot agree in the conclusion of the author, and we think that general experience plainly contradicts it. The muriatic, acetic, oxalic, tartaric, and lactic acids were found by him to prevent coagulation. The blood, in these experiments, was quite liquid. We are surprised that no mention is made of the remarkable effect of the vegetable acids, more especially the oxalic, on the colouring matter. This is assuredly the most striking and obvious change produced by these substances on the blood. Potash, ammonia, lime-water, the hydrosulphuret of ammonia, and nitrate of potash were then tried. All of these, with the exception of the hydrosulphuret of ammonia, were found to prevent the formation of a coagulum. Other substances were then mixed with recent blood, and the results noted. The carbonate and bicarbonate of soda prevented coagulation; the sesquicarbonate of ammonia gave no satisfactory result; the carbonate of potash liquefied the blood, but differed from that of soda, in turning the liquid black; (?) the sulphate of potash gave a precipitate of albumen and globules; the chloride of calcium, no appreciable effect; solution of chlorine gave a dark tint without coagulation; sulphate of iron produced an evident chemical reaction, throwing down albumen; alum prevented coagulation; (?) the bichloride of mercury and acetate of lead precipitated albumen; the ferrocyanate of potash entirely liquefied the blood; the nitrate of silver precipitated albumen; phosphate of soda gave a coagulum, as did likewise tartar-emetic; alcohol had but a slight effect,—hence in small doses, this liquid is not injurious; cinchonine, in the proportion of a grain, allowed a thin coagulum to form; sulphate of quinine, a scarcely perceptible coagulum; and a decoction of dried digitalis prevented coagulation. (p. 211 et seq.)

The author candidly acknowledges that this series of experiments is not complete, that the results in some instances have left doubts upon his mind, and that some of the experiments will require repetition. We are glad to find this acknowledgment made, because we cannot ourselves put any confidence in the greater number of the results. Thus the neutral sulphate of potash is represented as giving a precipitate of albumen and globules, while the acidulous solution of alum, contrary to the observations of most chemists, is laid down as preventing coagulation. What clear inference can be drawn from the addition of complex metallic salts to the blood? How can the effects of the nitrate of silver be compared with those of the phosphate of soda and sulphate of potash, when we know that the first-mentioned salt is so easily decomposed by all kinds of organic matter, and the last two are not? So again, who can suppose that the grain of cinchonine had any effect in producing the coagulum, which it is described to have done? We have thought it right to quote these experiments, because upon them the author appears to rest the originality and importance of his views. Our readers will perceive that he has not taken any pains in the performance of them, to avoid fallacies which must attend all such investigations. The peculiar effect of metallic salts is not allowed for, nor does it appear that specimens of blood were preserved *unmixed*, to compare with those to which the substances have been respectively added. Without this latter precaution, it is impossible to connect the result with the experiment so strictly as sound induction requires. We cannot join him in applying these results to the living system, nor can we agree that the results obtained in test-tubes on blood drawn from the body, are necessarily the same as those which follow when the substances are injected into the living vessels. In one part of his work, already quoted, he admits that the blood is different in properties in the two cases; but apparently forgetful of this statement he is continually applying his experiments out of the body, without the least discrimination, to those effects which take place in parts and organs endowed with life, and therefore under very different conditions.

Other experiments are related with other acids and salts; but these we do not consider it necessary to extract, since they are open to the same objections as the preceding. While boracic and arsenious acids liquefy the blood, he finds that borax produces a clot. On inspecting an animal which had died from an injection of citric acid into its veins, he discovered, exactly as he had anticipated, extravasation of blood and serum in the lungs. "It is true there was no decided congestion, but that was explained by the injection not having been sufficiently concentrated to put an immediate stop to the circulation." (p. 242.) Among the practical conclusions, we find the following. In order to determine in what dose sulphuric acid acts as a poison, he took eight equal portions of blood,—to the first he added one drop of the acid, to the second two, and so on with the rest. In the first vessel the blood was entirely liquid and incoagulable; in the third it was still more changed, and he felt confident "that an animal in whose veins such a liquid existed, would suddenly die." (p. 237.) "Again, hydrocyanic acid is one of the most violent poisons: six drops of it added to a certain quantity of blood, diluted with five parts of water, caused the globules and fibrine to disappear, an alteration in properties so great, that it was no longer surprising this poison should so rapidly destroy life." (p. 254.) These are specimens of the vague generalities in which the author frequently indulges!

We shall pass over the experiments relating to the influence of gases on the coagulation of the blood, and proceed to notice our author's views of the nature of the *buffy coat.* In various lectures we find this subject adverted to, and, in general, the remarks on it are accompanied by a sweeping condemnation of the modern doctrines of pathology. Magendie considers, and in our opinion correctly, that the fibrine and colouring matter are distinct principles in the blood, and that the buffy coat is nothing more than a layer of fibrine, coagulated without the red particles, containing commonly a portion of serum. The separation is due to the difference of density between the fibrine and red particles; but we do not agree with him in thinking that the greater density of the latter is to be ascribed to their containing iron. (p. 285.) The peculiarity of our author's views on this subject is, that he denies the connexion which pathologists have stated to exist between the formation of a buffy coat and the presence of inflammation. He denounces this as a chimerical notion, which it will require but a few years to banish entirely from the doctrines of the schools. The reasons for his opinion are well set forth. He has often looked for it, in cases where it might have been expected, without success: an analogous condition of fibrine frequently occurs in the coagulation of the blood of horses, without its being connected with any inflammatory condition of the body; lastly, its occurrence in human blood depends on numerous extrinsic circumstances, totally unconnected with disease, even where inflammation undoubtedly exists. Thus, if the opening made in a vein be too small, or if it be not parallel with that in the integuments, or if a globule of fat intervene, so that the blood froths in escaping, the buffy coat will not form on the coagulum. If a large opening be made, and the blood be received in a deep narrow vessel, it may assume in coagulating a buffy character. Hence, those who trusted in this sign, as indicative of inflammation, would meet with two opposite states of the blood in the same individual. If this were a true pathognomonic sign of inflammation, it ought to show itself in all similar cases, which it does not: but it is an accidental and unimportant condition, not at all fitted to become a guide for treatment. Why certain kinds of blood present this phenomenon and others do not, it is impossible, in the present state of science, to say. (p. 290.) These arguments of the author appear to justify the view which he takes; but we think experience has long ago fully established a connexion between this condition of the blood and an inflammatory state of the body; and, as he justly remarks, on another occasion, one fact cannot overthrow another, we would beg to ask, whether these apparently conflicting circumstances may not be reconciled. Thus we do not see that it is a necessary consequence of the general view, that the buffy coat must, as a matter of course, be present in every case of inflammation, or that it may not occur in other states of the system. Again we see no objection to the doctrine of the formation of this coat being modified by the manner in which the blood is drawn from the system; for physical circumstances out of the body may easily be conceived to favour or prevent it, nor can we admit it as an axiom that a condition of the solids or liquids, to be regarded as a pathognomonic character of a particular disease, should be universally met with. Certainly, if the author's distinction between a pathological and an accidental change were to be regarded as true, pathology would be reduced to a chaos of useless facts. In practice the author's fears as to the effects of the generally received doctrine are, we think, groundless; because a well-judging practitioner does not continue to bleed his patient from the mere occurrence of a buffy state of the blood, but he is guided by numerous other circumstances.

The author now passes to the consideration of the other principles of the blood, namely, the albumen and globules; and here we find more original and interesting matter than in the first part of the volume. The most striking difference between fibrine and albumen is that the latter does not possess the same property of spontaneous coagulation. Albumen may be solidified by heat and various reagents; but when thus coagulated, it does not present that filamentous network observed in coagulated fibrine. (p. 283.) Alcohol is well known to cause the coagulation of albumen, a circumstance which proves that alcoholic liquids should be taken in moderation. If a pretty strong dose of alcohol be given to an animal, it speedily dies, and albumen is found coagulated in its vessels. (p. 311.) (?)

Albumen coagulated by alcohol differs from that coagulated by heat, principally in the fact that it is in small flocculi; while albumen which has been heated is met with in large masses. In these remarks we must not confound the albumen of the egg with that of serum. These two liquids are distinguished from each other by some well-marked characters. If a small quantity of solution of potash be added to the albumen of egg, the whole becomes a transparent gelatinous mass, very much resembling pure gelatine in appearance. It is an albuminate of potash. On adding the alkali to the serosity of ascites, a slight precipitate was produced, but the mixture was quite liquid. (p. 312.)

It is singular that Magendie should have used the serosity of ascites, when immediately before he had been speaking of the serum of blood. In repeating this experiment, however, we have found that serum is merely diluted by the alkaline solution, while the albumen is gelatinized. In admitting this difference, we must remember that albumen of egg is much more viscid and firm in its ordinary state than serum. Potash in excess, it is to be observed, equally prevents serum and albumen from coagulating by heat, a point of resemblance not adverted to by the author, but which materially affects the results of some of his experiments.

The following differences are new to us:

Acetic acid, added to serum, produces no particular effect; added to albumen it causes an apparent partial coagulation, but which is due to a separation of the membrane of the cells. On boiling the two liquids there is a striking dissimilarity in the results. The acetate of serum becomes opaline from partial coagulation, but it remains liquid. On cooling it becomes opaque and solid; on reheating it, it again becomes liquid. The acetate of albumen is differently affected by heat; it forms a solid coagulum, separating almost entirely from the acetic acid, which remains transparent and slightly gelatinous. A continued application of heat does not reliquefy the coagula of albumen.

Pure ammonia, added to serum and albumen, produces no particular effect; when boiled, the ammoniacal serum does not coagulate, while the ammoniacal albumen swells up and coagulates into a light spongy mass. (p. 312.)

The lecture is here so badly reported that we have thought it better to give the author's meaning, than to adhere to a strict translation.

M. Magendie remarks that there is a striking difference between albumen of serum and fibrine, for many substances solidify albumen, while few have the property of liquefying it. It is just the reverse with fibrine.

Many other reagents which affect albumen are next pointed out. Ether is commonly said to distinguish albumen of egg from serum, because, while it coagulates the former entirely, it scarcely affects the latter. This is generally true; but we have met with specimens of serum in which ether, besides dissolving out the oil, produced a coagulum. Oil of turpentine does not, in our view, exert a different action on these two liquids, as it is often stated to do. It coagulates both, when the mixtures are well agitated. M. Magendie asserts that the sulphate of lime in solution coagulates albumen; and he proceeds to draw an inference therefrom on the use of hard water as a diet drink. (p. 323.) In repeating the experiment with a perfectly saturated solution of that salt, we did not find that it had any other effect than the addition of distilled water. It separated the membrane of the cells in loose flocculi, but nothing further. It merely diluted serum. The very imperfect solubility of this salt will easily explain why it should exert no action on these liquids.

The author considers that he has made an important discovery in the fact, that when albumen of egg is injected into the veins of a living animal, it is speedily and rapidly deprived of those characters by which he has shown it to be distinguished from serum; in short, he imagines that it is transformed into serum. The experiment is not unattended with danger to the animal. "The albumen of four eggs, carefully strained, was introduced into the jugular vein of an animal. It was immediately seized with vomiting, but it lived two days without much pain, and then the albumen of two eggs was injected: on the following day a similar quantity was introduced, and the animal died immediately." (p. 324.) "It was once bled a few minutes after the injection, and its blood separated, on standing, into a liquid and solid part: but the serum, mixed with potash, did not form a gelatinous mass like albumen; it remained perfectly liquid. Hence it is to be inferred that this substance (albumen ovi), in traversing the economy, is entirely deprived of its distinctive characters." (p. 325.) That the animal should have died from the repeated injections of a thick and tenacious substance like albumen, so different from serum in its physical properties, does not in the least surprise us; but that the albumen thus employed was actually transformed into serum is a statement which we do not think can be admitted, since, to warrant the conclusion, the author should have examined the whole of the blood, and not a few ounces. Other objections might be urged to this speculative view. For instance, how far is the action of the alleged distinguishing tests likely to be affected by the intermixture of the two substances in different proportions? Can they detect a small portion of albumen ovi in a large quantity of serum? If not, what becomes of the conclusion? The author partly furnishes us with the means of answering this question; and we shall quote the following paragraph to show that we are justified in asserting that his experiments are not always trustworthy.

"Here is another fact not less curious in support of what has been previously stated. Before the lecture, some human serum was mixed with $\frac{1}{10}$th of its volume of albumen ovi. My object was to determine whether we should have the same result as that observed in the living vessels (the transformation of albumen into serum); and, however extraordinary this may appear, it is undoubtedly the case. The albumen has disappeared, and the serum, treated by potash and heated, does not coagulate as the albumen ovi would do under the same circumstances. This appears to me to be a sufficiently convincing proof, from which we may conclude that the simple intermixture of serum with albumen ovi deprives this last-mentioned substance of the property of coagulating by heat." (p. 332.)

We have looked among the errata for some explanation respecting this paragraph; but finding none, we must charitably suppose that the experiment was hastily performed, and the conclusion inconsiderately drawn. We think it our duty to point out the error into which, we conceive, the Professor has inadvertently fallen. Potash, added to albumen or serum in sufficient quantity, prevents either liquid from coagulating by heat; and the presence of $\frac{1}{10}$th of albumen in serum can of course make no difference in the action of the alkali. Possibly the author may have intended ammonia for potash; but if we allow the substitution, it will assist him but little in his inference; since we cannot expect that the action of this alkali, aided by heat, should be very different on a liquid which was pure serum, or on one of which nine tenths were serum. The pretended transformation of albumen into serum, is as little established by these experiments out of the body, as by those which were performed on a living animal.*

We are glad to find that the opinion which we expressed in our Twelfth Number, relative to the experiments of M. Denis, on fibrine, is fully borne out by the result of their repetition before M. Magendie. Our author states, and we think justly, that there are not sufficient reasons for believing albumen and fibrine to be one and the same substance slightly modified. M. Denis's opinion of their being identical was chiefly founded on the statement "that fibrine, digested in nitrate

* Since the preceding observations were written, we have had occasion to repeat some of M. Magendie's experiments, particularly those on *serum* and *albumen*, and are now in a condition to speak positively on the subject; we think it, however, better to let the remarks stand in the text as they were originally written.

M. Magendie states the differences of albumen and serum as follows: 1. Potash forms a jelly with *albumen*, not with *serum*. 2. On ammonia being added to *albumen*, and the mixture boiled, the whole forms a solid spongy coagulum: on ammonia being added to *serum* and boiled, no coagulum whatever is produced, the whole remains liquid. 3. Acetic acid boiled with *albumen* forms a thick coagulum: with *serum* it forms, on boiling, an opaline liquid, becoming nearly solid on cooling, again liquid on heating, &c.

Now, of these assumed differences, we find that the first two are dependent entirely, as we suspected, on the different degrees of viscidity and consistency in the pure albumen ovi and serum; so that if the albumen be diluted with water to the consistency of serum, potash and ammonia act precisely in the same way on the two liquids. The effect of acetic acid and heat on serum and diluted albumen is different. Acetic acid does not prevent the coagulation of diluted albumen by heat, but its effect with serum is as above stated (3); hence this is the only true chemical difference.—REV.

of potash, becomes dissolved, and then acquires all the properties of albumen: among others, that of becoming coagulated by heat." The author shrewdly observes: "M. Denis, who is one of my colleagues, came to announce this fact to me; but, as I prefer seeing things with my own eyes, and not with the eyes of others, I begged him to repeat his experiments before me. They were accordingly performed in this very theatre before many of you; but unfortunately not one of the results announced was obtained." (p. 331.)

We have already observed that M. Magendie regards the *globules* of blood as distinct from fibrine. He considers that of all the constituents of this liquid, these undergo the least change in severe diseases. In the dead it has been remarked that the membrane which forms the envelope appears shrivelled and contracted, in which condition M. Donné considered that there existed a well-marked sign of death. But M. Magendie established that the same condition of the globules existed in the blood of a healthy living man, when they had been allowed to remain for some time in a vessel. (p. 347.)

The following remarks are to us new with regard to the globules of blood: but we strongly doubt whether the so-called animalcules really deserve the title.

"When the globules have become shrivelled on the surface, a number of infusoria (vibriones) show themselves in the serum. They give rise to vibratory and other motions of these globules, by striking against them. After a time they actually destroy their substance, and reduce them to opaque, indeterminate masses. Fresh globules introduced into serum, containing infusoria, were speedily attacked and destroyed by these animals. They may be usefully employed in the interests of science, by placing globules among them, since they cause these bodies to revolve in all directions, and thus favorably present themselves for observation." (p. 357.)

We find nothing further in a chemical or philosophical view, in relation to the globules of blood, but what our readers must be well acquainted with. One curious fact is mentioned, with regard to cutaneous absorption, and the transmission of substances into the circulation; namely, that "if the hands be rubbed with ether, the pulmonary exhalation will have an ethereal smell for one or two hours." (p. 386.)

The last lecture is devoted to a general summary of the objects of the course, in which the author indulges in some sarcastic reflections on the tedious discourses delivered in the modern medical schools of Paris, London, and Copenhagen, on the nature of chronic and acute diseases. He inveighs against pathology and pathologists of all classes, and often in very bad taste, (pp. 71, 196, 354;) but we think that this proceeds simply from overstrained enthusiasm in his subject. He is very fond of quarrelling with a name—witness the word *inflammation;* but he should remember that so long as practitioners understand each other, the name attached to a morbid condition of the body is a matter of little moment. We see in M. Magendie an ardent physiologist, devoted to the illustration of his subject by all kinds of experiments, many of which lead to no practical end; and the results of others, if not conflicting, appear to us misinterpreted. He is by no means sufficiently cautious to avoid those fallacies which attend every experiment on the *living* body; and he is too ready to infer from a result on the dead what must be the effect on the living. This we are not surprised at, when he seems to banish the idea of vitality from his mind, and to look upon the living body as little else than a machine, ruled almost entirely by physical laws. If his opponents have assigned too much to the influence of a vital principle, it is certain that he has assigned too little.

. We give M. Magendie every credit for ingenuity and a sincere devotion to his subject; but we have felt ourselves constrained to denounce what we considered to be erroneous in his experiments and reasoning. " Reasoning alone," he observes, at the conclusion of one of his lectures "can do nothing,—I am wrong—it may embarrass and obscure everything. Experiment, on the contrary, which is nothing more than the true method of reasoning correctly, can alone extricate us from a difficulty." (p. 339.) This observation is true to a certain extent; but we must remark that the falsest theories may be based upon experiments when the results are not rightly understood or are wrongly interpreted. Correct reasoning alone can guard us against this.

We here conclude our analysis of these singular volumes, which, whatever may be their influence on the progress of physiology, certainly deserve a place among the curiosities of medical literature. After the view we have taken of their contents, it is hardly necessary to say that we do not think they are likely to add to M. Magendie's reputation; nay, we must further express our conviction that if that reputation were not so firmly and justly established by his antecedent labours, such a production as the present would inevitably be fatal to it. Science, doubtless, stands indebted to M. Magendie for the bold and distinct announcement of the truth that many of the phenomena of life are simply physical, and should be investigated by the application of physical laws; but here his merit ends. Instead of carefully laying the foundations of a new branch of science, he has reared on the sand a frail and fantastic edifice, which crumbles away even under the hands of its architect.

On a sober view of the subject, it is evident that, in order to pursue with success the study of the physical phenomena of life, we should commence by a minute and laborious investigation of the physical properties of all the textures of which living bodies are composed; the relation of these textures to all the physical agents capable of effecting changes in them; their varieties at different ages and in different orders of animals, and the diversity of their properties in life and in death. How little is known on any of these heads! If so, the study of the physical phenomena of life is yet to be begun. Its philosophical cultivation could not fail to yield the most interesting results; many long-known physical truths would be placed in new points of view, and endowed with increased applicability; many sterile tracts of science would be fertilized, and many new relations established, from which physiology and physics would derive splendid and reciprocal light.

